# Integrating *Drosophila* and *Vibrio fischeri* models for toxicity evaluation: uncovering detoxification trends in psoralea Fructus-TCM formulations

**DOI:** 10.3389/fphar.2025.1590929

**Published:** 2025-06-13

**Authors:** Cheng Zhang, Yina Li, Fangyang Li, Wanyun Dang, Zhuo Shi, Chunqi Yang, Chengrong Xiao, Xianglin Tang, Yuguang Wang, Yue Gao

**Affiliations:** ^1^ School of Pharmacy, Guangdong Pharmaceutical University, Guang zhou, China; ^2^ School of Life Sciences, Hebei University, Baoding, China; ^3^ Beijing Institute of Radiation Medicine, Beijing, China; ^4^ Faculty of Environment and Life Science, Beijing University of Technology, Beijing, China; ^5^ National Key Laboratory of Kidney Diseases, Chinese PLA General Hospital, Beijing, China

**Keywords:** psoraleae fructus, detoxification in combination with TCM, toxicity detecting, toxic components screening, *Drosophila melanogaster*, *Vibrio* fischeri

## Abstract

**Background:**

The toxicity of herbal medicine combinations is critical to the clinical safety of traditional Chinese medicine (TCM). Current assessment methods are often inefficient and costly, creating an urgent need for new strategies to evaluate herbal medicine toxicity. We conducted research based on the commonly used TCM, Psoraleae Fructus (PF), and its formulations, Er Shen Pills (ESP) and Si Shen Pills (SSP).

**Methods:**

We conducted a series of analyses on *Drosophila*, including survival analysis, enzyme assays, and quantitative PCR(qPCR) tests, to evaluate the effects of various TCM combinations on fruit fly health and viability. Transcriptome sequencing was utilized to investigate the detoxifying mechanisms of these combinations. Additionally, experiments with *Vibrio fischeri* assessed toxicity changes by calculating the luminescence inhibition rate. An innovative similarity model was developed to identify toxic components within the TCM formulations. Finally, molecular docking and molecular dynamics simulations explored the mechanisms of action of these toxic components on *Vibrio fischeri*, providing a comprehensive understanding at the molecular level.

**Results:**

In *Drosophila* experiments, ESP and SSP groups showed longer survival times, with male flies being more sensitive, making them more suitable for toxicity studies. Enzyme assays indicated a decreasing toxicity trend for ESP and SSP compared to PF, with significant changes observed in female flies. The qPCR analysis revealed that the upregulation of *cpr* and *cyp6a8*, along with the downregulation of *keap1*, *hsp22*, *hsp68*, *gstD6,* and *hsp83,* can assess the toxicity changes of PF, ESP, and SSP. The primary detoxification pathway involves the metabolism of xenobiotics by cytochrome P450. In the *Vibrio fischeri* assay, the IC_50_(50% inhibition) value of ESP was the highest, indicating reduced toxicity compared to PF. Screening for toxic components revealed that PF had 4, ESP had 16, and SSP had 22 components, primarily acting on LuxD, LuxE, and LuxG enzymes.

**Conclusion:**

A method for detecting the toxicity variation patterns of PF, ESP, and SSP can be established using *Drosophila* and *Vibrio fischeri*, and the mechanisms of toxic effects can be explored respectively through transcriptomics and virtual screening techniques.

## 1 Introduction

The concept of combination in Traditional Chinese Medicine (TCM) is well-documented, dating back to the *Huangdi Neijing* (written between 475 B.C. and 26 A.D.), which outlines the Prescription Principles: sovereign, minister, assistant, and guiding. Similarly, the *Shanghan Zabing Lun*, a classical text from the Han Dynasty, includes numerous examples of mutual combinations used to mitigate toxicity (Liu et al., 2017). The strategic combination of Chinese medicine prescriptions forms the foundational framework for their clinical application, as an effective combination may achieve detoxification or enhance therapeutic efficacy through ingredient interactions (Guo et al., 2012).

After combining TCMs, researchers typically use cell experiments and rodent animal studies, both *in vitro* and *in vivo*, to verify toxicity levels and changes. However, these methods are time-consuming, inefficient, and costly. Thus, accurately, quickly, and efficiently evaluating the toxicity of TCM combinations remains a critical challenge in TCM toxicity research. To address this, innovative pharmacological methods are necessary.

Using *Drosophila melanogaster* and *Vibrio fischeri* for toxicity detection belong to New Alternative Methods (NAMs). In addition, organisms such as zebrafish (Chahardehi et al., 2020) and *Caenorhabditis elegans* (Boyd et al., 2012), along with *in silico* screening models like Quantitative Structure - Activity Relationship (QSAR) (Yang et al., 2021), 3D-QSAR(Jiang and Gao, 2018), other relevant screening models (Jia et al., 2025) etc., and molecular docking techniques (Yang et al., 2022) are all highly promising methods for toxicity detection.

We selected commonly used clinical combinations of TCMs, such as Psoraleae Fructus (PF), to explore new approaches using the *Drosophila melanogaster* and *Vibrio fischeri* model organisms. *Drosophila melanogaster* offers several advantages: it is inexpensive, easy to maintain, reproduces quickly, and has a well-documented genetic background (Reaume and Sokolowski, 2006; Rubin, 1988). Although it has been utilized in pharmacological research to assess the treatment effects of TCM (Cao et al., 2022; Teseo et al., 2021; Yu et al., 2024). Moreover, in the research of the toxicology of TCM, there have also been reports that fruit flies are used to study genotoxicity (Abraham et al., 1978; Pimenta and Nepomuceno, 2005), developmental toxicity (Jun et al., 2012; Kushalan et al., 2022), etc., which play an important role in promoting the evaluation of the toxicity of TCM. its application in evaluating the toxicity of TCM combinations, particularly concerning building methods, is limited.

*Vibrio fischeri*, a marine photobacterium, is extensively used for toxicity monitoring due to its high sensitivity, time-efficiency, cost-effectiveness, and straightforward operation (Abbas et al., 2018; Su et al., 2023; Sun et al., 2023). A Chinese research team developed a biological testing method based on MicroTox technology, which utilizes the luminescence intensity of bioluminescent bacteria to study the toxicity of TCM (Xiong et al., 2016). This method combines the unique physiological characteristics of luminescent bacteria with modern photoelectric detection technology to detect toxic substances rapidly.

Given the high cost, time consumption, and large sample requirements of animal and cell testing, recent studies have emphasized the benefits of rapid, reproducible, and cost-effective bacterial detection for toxicity screening and evaluation. Our research focuses on toxic influence in combination with TCMs in *Drosophila melanogaster* through the changed enzyme, gene and life spans expressed *in vivo* experiments to establish the detecting toxicity paradigm. At the same time, *Vibrio fischeri* will be nailed to rapid toxicity testing and screening of toxic components to build the testing toxicity protocol. A new method to test the toxicity of a combination of TCMs has been made.

## 2 Materials and methods

### 2.1 Processing methods of a combination of TCM

Psoraleae Fructus (PF) (*Psoralea corylifolia* L.) is combined with different TCMs to formulate Ershen Pills (ESP)and Sishen Pills (SSP). Both formulations are commonly used in clinical practice as aqueous extracts. PF aqueous extracts are prepared from salt-processed PF. ESP consists of PF, Myristicae Semen (Myristica fragrans Houtt.) (dried ripe fruit), Fresh Ginger (*Zingiber officinale* Roscoe) (fresh root), and Jujube Dates (*Ziziphus jujuba* Mill.) (dried ripe fruit). SSP includes PF and Schisandrae Chinensis Fructus (*Schisandra chinensis* (Turcz.) Baill.) (dried ripe fruit). These formulas are composed according to the proportions in Chinese Medicine Formulas (Ji, 2014) and processed to conform to the Pharmacopoeia of the People’s Republic of China 2020 Edition (Committee, 2020) (Supplementary Material 1). The preparation process involves soaking the ingredients in ultra-pure water at ten times their volume for 8 h to ensure adequate hydration. After soaking, the mixtures are boiled twice, each time for 1 h. Following boiling, the solutions are filtered and converted into lyophilized powder for storage at −20°C. The plant names listed above were verified on World Flora Online (http://www.worldfloraonline.org) as of August 13, 2024. The proportions of the compound medication are available in Supplementary Material 1. Additionally, the chemical fingerprints, the ratio of the drug to the extract, as well as other basic pharmaceutical parameters for each test extract are elaborated in our previously published article (Zhuo Shi et al., 2024).

### 2.2 Fly stock and culture

Wild-type *Drosophila melanogaster* was used Canton-S Strain (Fungene biotech, China; From RaoYi lab) in all experiments. Flies were maintained on plastic vials containing Control or TCM culture medium, in a 24°C environment with 12 h day-night-shift. The Control culture medium contains 52.5 g cornmeal, 27.5 g sucrose, 20 g yeast, 8 g agar, and 0.25 mL propanoic acid with 500 mL ultra-pure water (Shen et al., 2012). The TCM culture medium is prepared by adding lyophilized powder on the basis of the control culture medium. The amount of lyophilized powder to be added is calculated according to the yield rate of the TCM extract (Supplementary Material 1). To investigate the toxicity variation trends of PF when combined with different TCMs to formulate ESP and SSP, the culture medium is required to contain 0.04 g of the original medicinal material of PF per milliliter (The 0.04 g dose was determined based on evaluation and analysis following a 24-h acute toxicity test in *Drosophila melanogaster*). 3.39 g of the lyophilized powder of PF needs to be added for PF group, 8.72 g of the lyophilized powder of Ershen Pills (ESP) is added to the ESP group, and 14.95 g of the lyophilized powder of Sishen Pills (SSP) is added to the SSP group.

### 2.3 Lifespan assays

Newly eclosed fruit flies were sorted by sex within 3 days post-eclosion. Thirty fruit flies were placed in each culture vial, with each experimental group comprising 300 flies (10 vials per group). The vials with culture medium for fly maintenance were replaced every 3 days for the first month. After the initial month, the frequency of the procedure should increase to every 2 days. At the same time, flies death number was recorded each day. During the replacement of culture vials, some flies (≤7%) escaped and died accidentally, which has been excluded from the calculation. IBM SPSS Statistics 27 was used to analyse the data and the Kaplan-Meier survival curve was drawn by Bioinformatics (https://www.bioinformatics.com.cn/).

### 2.4 Enzyme test

Newly eclosed fruit flies were sorted by sex within 3 days post-eclosion. Sixty flies were placed per culture vial, and the culture medium was replaced every 3 days. After 7 days, 0.1 g of the sample was collected for analysis and stored at −80°C. For testing, 0.1 g of the sample was homogenized in an ice bath with 1 mL of extraction buffer and then centrifuged at 8000 *g* for 10 min at 4°C. The supernatant was collected for further tests. Acetylcholinesterase (AchE), Carboxylesterase (CarE), and Glutathione S-transferase (GST) activities were measured using a Biotek Cytation5 (BioTek Instruments, Inc.) following the manufacturer’s instructions provided in the Activity Assay Kit (lot numbers BC 2025, BC0845, BC0355, Solarbio Science & Technology Co., Ltd., Beijing).

### 2.5 Quantitative real-time PCR

The preparation of testing samples was identical to that used for the enzyme tests. Each sample underwent three biological replicates. Due to the differential expression of sex-biased endogenous genes in response to external stressors, we compared the stability of *RP49*, *RP49_66*, *act5c*, *α-tub84b*, and *rps20* (Supplementary Material 2). Ultimately, the housekeeping gene rps20 was chosen for normalization. Relative expression levels of genes was calculated using the 2^−ΔΔCT^ method (Arocho et al., 2006).

### 2.6 Transcriptome sequencing

The preparation of the testing samples was the same as for the Enzyme Test. Using eukaryotic transcriptome sequencing with *Drosophila* as the reference species, all mRNA transcribed after administering the TCM to the fruit flies for 7 days was studied on the Illumina/MGI sequencing platform (Genewiz Biotech Co., Ltd., Suzhou). In the experiment, we used 12 samples of female *Drosophila* and 12 samples of male *Drosophila* respectively. In addition, the Control group and each TCM group were repeated biologically 3 times to ensure accuracy and consistency. The clustered heat map was drawn by Pheatmap in R software based on FPKM (Fragments Per Kilobase of transcript per Million mapped reads). Based on a p-value less than 0.05 and |Foldchange|≥2, volcano plot was created. GOSeq (v1.34.1) was used to identify Gene Ontology (GO) terms that annotate a list of enriched genes. Significant differential expression genes were enriched in KEGG (Kyoto Encyclopedia of Genes and Genomes) pathways.

### 2.7 *Vibrio fischeri* toxicity assays

TOX-kit-100F *Vibrio fischeri* (NRRL B-11177) (Bixiao Environmental Technology Co., Ltd., Hunan) was used to detect toxicity by luminescence intensity in Biotek Cytation5 (BioTek Instruments, Inc.). It complies with ISO 11348 standards and conducted the acute toxicity tests based on the guideline of Determination of the Acute Toxicity-Luminescent Bacteria Test (GB/T15441-1995). According to the luminescence principle of *Vibrio fischeri* build the new protocol as a reference based on TOX-kit-100F instruction. The 96 micro-porous plate (Costar 3599) is the experimental carrier. Firstly, from the TOX-kit-100F, take one tube of bioluminescent bacteria, add 3 mL of bacterial reactivation solution, gently shake until evenly mixed, and let it sit for 5 min. Secondly, take 180 µL of the test sample and Control (ultrapure water) respectively, add them to a 96-well plate, then add 18 µL of osmotic pressure adjustment solution (10:1 ratio) and mix well (repeat four times for each concentration). Then, take 20 mL of the revived bacterial solution and add it to the test wells. Finally, after 15 min of adding the sample, measure the bioluminescence intensity, then calculate the inhibition rate using the formula:

$$\text{Inhibition}\left(\%\right)=\frac{\vec{Χ}\text{ctrl}\;{\text{l}}_{\text{um}}-\vec{Χ}\text{sample}\;{\text{l}}_{\text{um}}\;}{\vec{Χ}\text{ctrl}\;{\text{l}}_{\text{um}}}\times 100\%$$

For the sample concentration gradient settings, refer to (Table 1), GraghPad prism 9 was used to analyse IC_50_ and draw graphics.

**TABLE 1:** The sample concentration for toxicity testing.

TCM	1 (g/mL)	2 (g/mL)	3 (g/mL)	4 (g/mL)	5 (g/mL)	6 (g/mL)	7 (g/mL)
PF	2.950 × 10^−4^	4.425 × 10^−4^	5.900 × 10^−4^	1.475 × 10^−3^	2.950 × 10^−3^	4.425 × 10^−3^	5.900 × 10^−3^
ESP	1.720 × 10^−4^	2.294 × 10^−4^	5.733 × 10^−4^	1.147 × 10^−3^	2.294 × 10^−3^	3.440 × 10^−3^	4.587 × 10^−3^
SSP	1.003 × 10^−4^	1.338 × 10^−4^	2.007 × 10^−4^	2.676 × 10^−4^	3.344 × 10^−4^	4.682 × 10^−4^	6.689 × 10^−4^

### 2.8 Toxic components screening

Constructing a toxicity molecular similarity model based on MACCS(Molecular ACCess System). Firstly, 1277 toxic molecules were dug from published open datasets (https://github.com/hhaootian/toxicity/blob/main/src/clean.py) (Ketkar et al., 2023). MACCS was applied using OpenBabel (https://github.com/openbabel/openbabel/releases), which was set up before the use of the MACCS. Secondly, Python 3.12.3 was used to run the MACCS featurization script (https://github.com/ZhangChengCADEN/MACCS-Model) for matching similarity between molecules when 1277 toxic datasets were finished to format, and toxic molecules, which are relatively strong, were obtained if the Tanimoto coefficient is greater than or equal to 0.5. In the end, molecules with strong correlation coefficients were analyzed for their Degree values using CytoNCA in Cytoscape (https://cytoscape.org/). Those with a Degree value of greater than or equal to 90 were further selected. The finally obtained molecules were used to construct a similarity model for toxic molecules. Components of PF, ESP, and SSP were obtained from the TCMSP database (https://www.tcmsp-e.com/) and were converted into SMILES format. A previously constructed similarity model was utilized to analyze the similarity between the TCM components and the toxic components. Following specified conditions, such as a Tanimoto coefficient greater or equal to 0.5 and a Degree greater or equal to half of the maximum value, the toxic components of TCM were successfully screened out.

### 2.9 Prepration of *Vibrio fischeri* LuxCDABEG luminescence enzymes

The luminescence enzymes LuxA, LuxB, LuxC, LuxD, LuxE, and LuxG collectively regulate the light emission in *Vibrio fischeri*. LuxA and LuxB encode the α and β subunits of the luminescence enzyme, respectively. Together, they form the LuxAB heterodimer, which is capable of producing light in bacteria. According to published articles on *Vibrio fischeri* species and protein structure resolution, LuxAB (3FGC) and LuxG (1BKJ) are the best choices from the RCSB Protein Data Bank (RCSB PDB) (https://www.rcsb.org/). However, they are not available in the RCSB PDB through a protein prediction database. LuxC (A0A510UCI8), LuxD (A7MAR5), and LuxE (P24272) were obtained from the AlphaFold Protein Structure Database (https://alphafold.com/).

### 2.10 Docking analysis

Schrödinger Maestro 2021 software (https://www.schrodinger.com) was used for docking analysis. Initially, the molecules were processed using LigPrep for OPLS4 field treatment, limiting the ligand size to fewer than 500 atoms, and Epik was used for ionization treatment. The Protein Preparation Wizard was then employed for pre-treatment before protein docking. During the H-bond assignment, PROPKA was used for optimization, and the OPLS4 field was chosen to minimize all atoms. Next, a docking box was constructed using Receptor Grid Generation, employing the “picking to identify the ligand” method to build the docking box for LuxA, LuxB, and LuxG. Blind docking was used for LuxC, LuxD, and LuxE because these proteins required ligands for action during structural measurement, thus bringing their ligands, while the latter was predicted by AlphaFold and did not have ligands present. Finally, molecular docking with proteins was performed using Standard Precision (SP). After docking was completed, the scores from molecular and protein binding were exported. Bioinformatics tools were then used for visual cluster analysis of the molecules and docking scores (Clustering method: complete, Distance method: Euclidean, Callback function: pheatmap).

### 2.11 Molecular dynamics analysis

Molecular dynamics simulation uses GROMACS 2020 (http://www.gromacs.org; Lindahl et al., 2020), and compilation is required before use. Step1. Separating the protein and molecular complex post-docking, using Pymol (https://pymol.org). Step2. Utilizing ATB (https://atb.uq.edu.au; Malde et al., 2011) to add force fields to the molecule and installing the corresponding force field package into GROMACS. When processing the protein, ensure that the ligand molecular force field and the protein force field are consistent. After the force field is added to the molecules, check whether hydrogen has been added; if not, it needs to be added. Step3. Invoke GROMACS command to convert the ligand’s molecular format into gro format, build the topology of the protein, ensure that water molecules have been removed from the protein in advance, and then construct the complex topology file of the ligand and protein. Step4. According to the size of the complex, determine the size of the molecular dynamics simulation box, and then add solvent water to the box. Step5. Bio-system modelling, adding ions to the complex, minimization of energy, NVT thermal equilibrium, NPT pressure equilibrium, and density analysis steps, and data visualization with DuIvyTools (https://github.com/CharlesHahn/DuIvyTools/tree/dev/DuIvyTools; CharlesHahn, 2024). Step6. Invoke GPU for computation, and perform molecular dynamics simulation on the well-constructed bio-system. Analysis of the simulation results mainly focuses on the Root Mean Square Deviation (RMSD) stable state, calculation of Root Mean Square Fluctuation (RMSF), and calculation of gyration radius (Rg). Based on RMSD and Rg a free energy landscape profile map is drawn by Origin 2021.

## 3 Results

### 3.1 The slowing of *Drosophila* death rate by Ershen Pills and Sishen Pills in the survival duration determination

**FIGURE 1:**
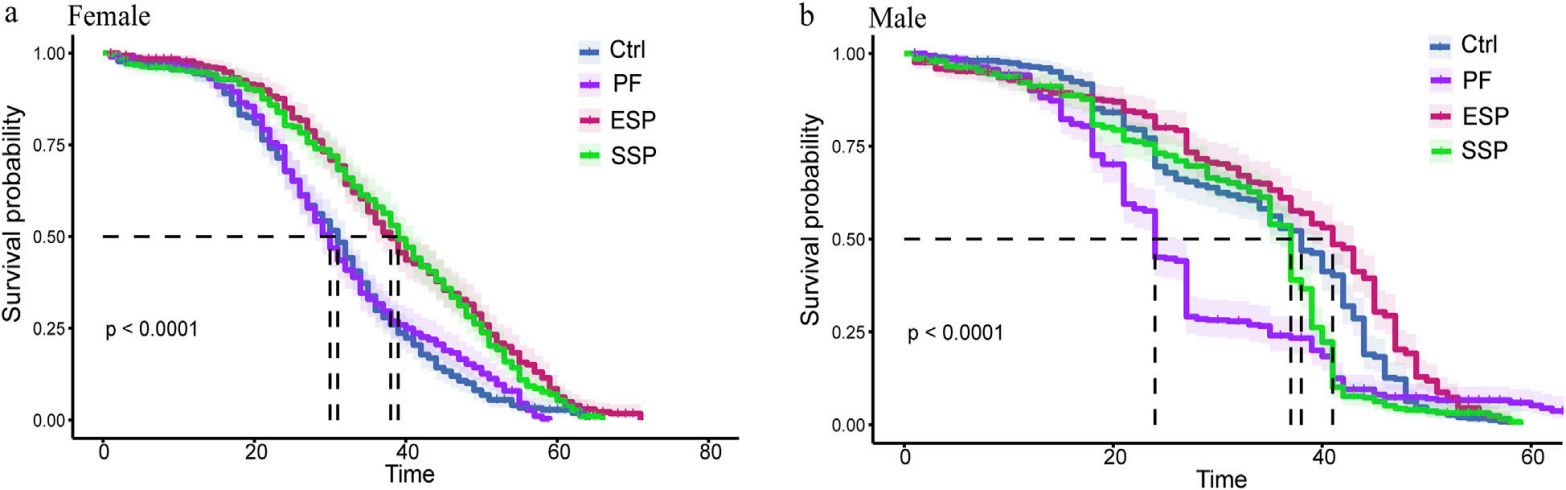
*Drosophila* Kaplan-Meier (K–M) curve based on TCM administration in female and male. **(a,b)** In Female and male *Drosophila* K-M curve of PF, ESP and SSP administration and the curve colors correspond to the TCM name.

In female fruit flies, The 50% survival rate (Median) order of variation was SSP > PF(p < 0.001), ESP > PF(p < 0.001), Control > PF(p = 0.581) (Table 2a), with the longest survival timing of the ESP group being 71 days, and the shortest of the PF group clocking in at 59 days (Figure 1A). From these survival times in female fruit flies, it can be seen that the same PF dose presented a lower toxicity trend after forming SSP and ESP. In male fruit flies, The 50% survival rate (Median) alteration was ESP > PF(p < 0.001), SSP > PF(p = 0.002), Control > PF(p < 0.001) (Table 2b), with the PF group presenting the longest survival time of 67 days and the shortest being the ESP group at 58 days (Figure 1B). From the survival times of male fruit flies, It can be inferred that the detoxification trend was occurred when PF is combined with other TCM to form SSP and ESP.

**TABLE 2:** The Kaplan-Meier analysis of *Drosophila* lifespan.

a. Female			
Group	Median (Days)	Mean (Days)	Log rank significance
			PF
Control	31.000	31.114	0.581
FP	30.000	31.769	—
ESP	38.000	39.333	<0.001
SSP	39.000	38.411	<0.001

b. Male			
Group	Median (Days)	Mean (Days)	Log rank significance
			PF
Control	38.000	34.230	<0.001
PF	24.000	27.266	—
ESP	41.000	36.665	<0.001
SSP	37.000	32.145	0.002

### 3.2 The reduction by Ershen Pills and Sishen Pills of the inhibition of AchE and CarE and the induction of GST in *Drosophila* caused by PF

**FIGURE 2:**
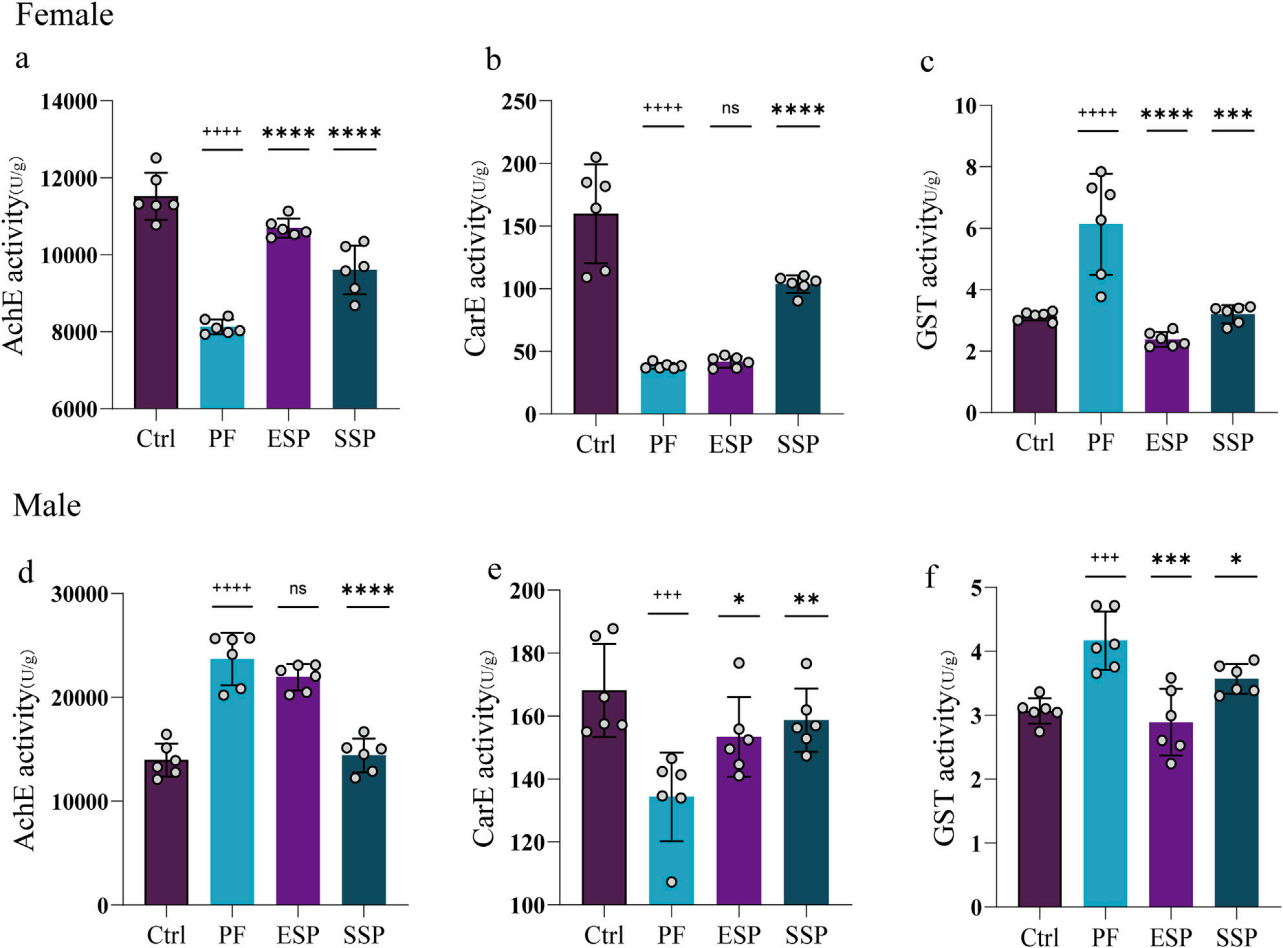
PF, ESP and SSP administration in female and male Drosophila affect on the change of (**a-d**) AchE, (**b-e**) CarE and (**c-f**) GST enzymes.^+^ indicates a significant difference compared to the Control, p < 0.05 (^++^p < 0.01,^+++^p < 0.001,^++++^p < 0.0001), *indicates a significant difference compared to the PF, p < 0.05 (**p < 0.01, ***p < 0.001, ****p < 0.0001), ns indicates no significant difference.

In studying the treatment of ESP and SSP at the same dosage as PF, in female and male fruit flies, it was found that the PF group, compared to the Control group, exhibited an inducing effect on GST. Simultaneously, ESP and SSP recorded a reduced inducing effect compared to PF (Figures 2C,F). Upon examining CarE, the PF group, compared to the Control group, showed an inhibitory effect on CarE(Figures 2B,E). However, the ESP and SSP groups reported a decrease in inhibitory effects compared to PF, with significant differences shown in male fruit flies (Figure 2E, p < 0.05, p < 0.01). In female fruit flies, only SSP showed a significant difference (Figure 2B, p < 0.0001), indicating that PF has inhibitory effects, while in males, ESP and SSP can alleviate these effects, but in females, only SSP exhibits this effect.

Investigating AchE, it was found that the PF group, compared to the Control group, showed inhibitory effects on the enzyme in females and ESP and SSP reduced this inhibition (Figure 2A, p < 0.0001). In males, the PF group was found to induce the enzyme compared to the Control group, and the SSP reduced this induction (Figure 2D, p < 0.0001). This implicates that, in female fruit flies, compared to ESP and SSP can reduce the inhibitory effect on AchE, while in males, only SSP can reduce the stimulatory effect on AchE. To summarize, in females and males SSP compared to PF, in reducing the inhibition of AchE and CarE and the induction of GST, shows a detoxifying trend, while ESP in female fruit flies, compared to PF in reducing the inhibition of AchE and the induction of GST, exhibits a detoxifying effect. In male fruit flies, a detoxifying trend is observed in reducing the inhibition of CarE and the induction of GST.

### 3.3 The upregulation of *cpr*, *cyp6a8,* and the downregulation of *keap1, hsp22, hsp68,* and *gstD6* in females and the downregulation of *hsp68, hsp83* in males can display trends of detoxification PF of ESP and SSP formulations

Clustering analysis: Through heatmap clustering analysis of 23 genes related to oxidative stress, toxic metabolism, etc., it can be seen that the three biological replicates are quite good. In females, each group of clusters are grouped together, and in males, most are classified together (Supplementary Material 3).

**FIGURE 3:**
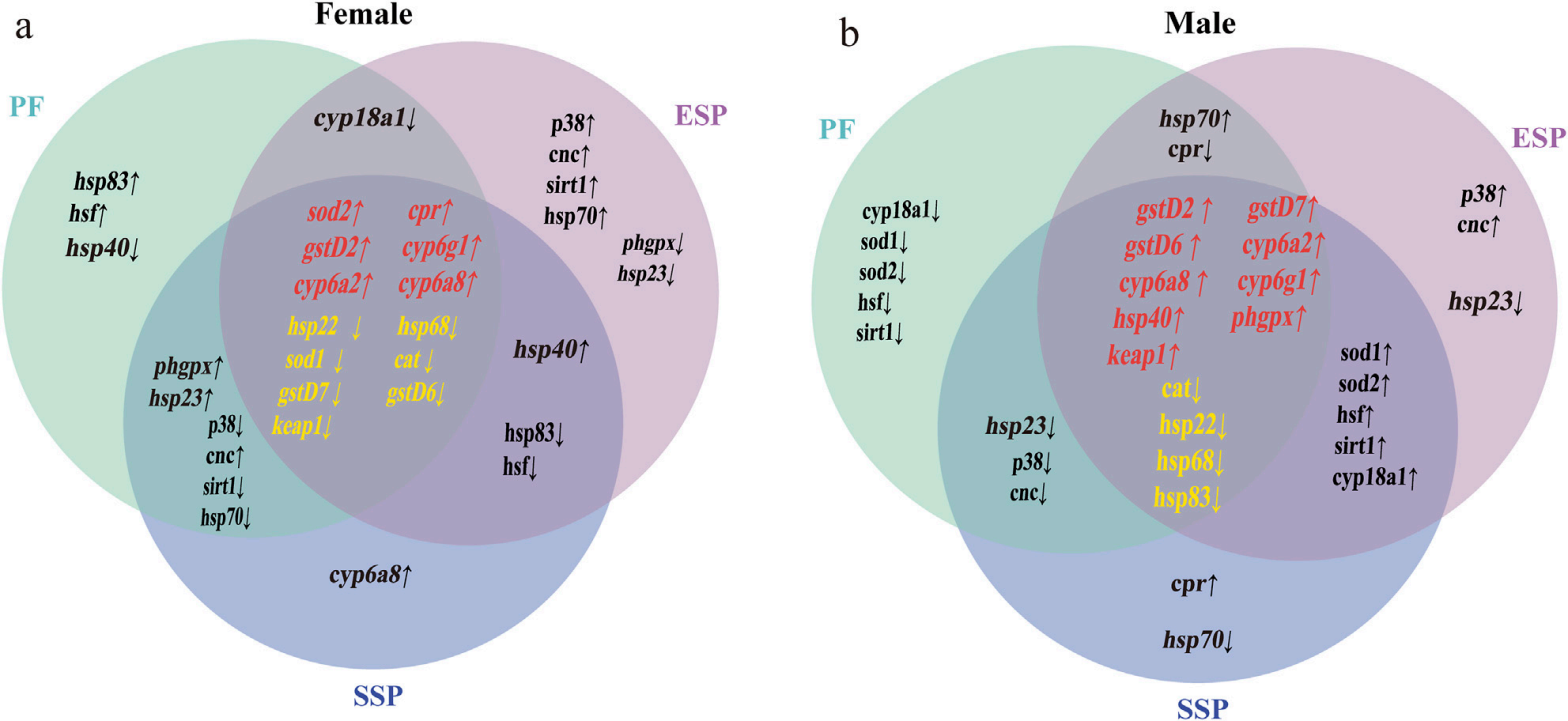
Up and downregulated co-expressed genes in Control, PF, ESP, SSP groups. In the Venn diagram **(a,b)**, the intersection part in the middle, genes marked in red indicate the co-expressed upregulated genes in PF, ESP, SSP, genes marked in yellow indicate co-expressed downregulated genes. The intersection parts on the left and right represent the co-expressed up and downregulated genes in PF and SSP, ESP and SSP. The intersection part above represents the co-expressed up and downregulated genes in PF and ESP.

Gene co-expression analysis: To more accurately express the changes in up-and-down regulated genes in each group and the co-expression of genes after drug combination, the average gene expression of three biological replicate samples was taken, and a Venn diagram of differential gene expression was made (Figures 3A,B). Through further analysis and screening of co-expressed genes, stably expressed genes were selected to evaluate the detoxification trend of Chinese medicine combination.

**FIGURE 4:**
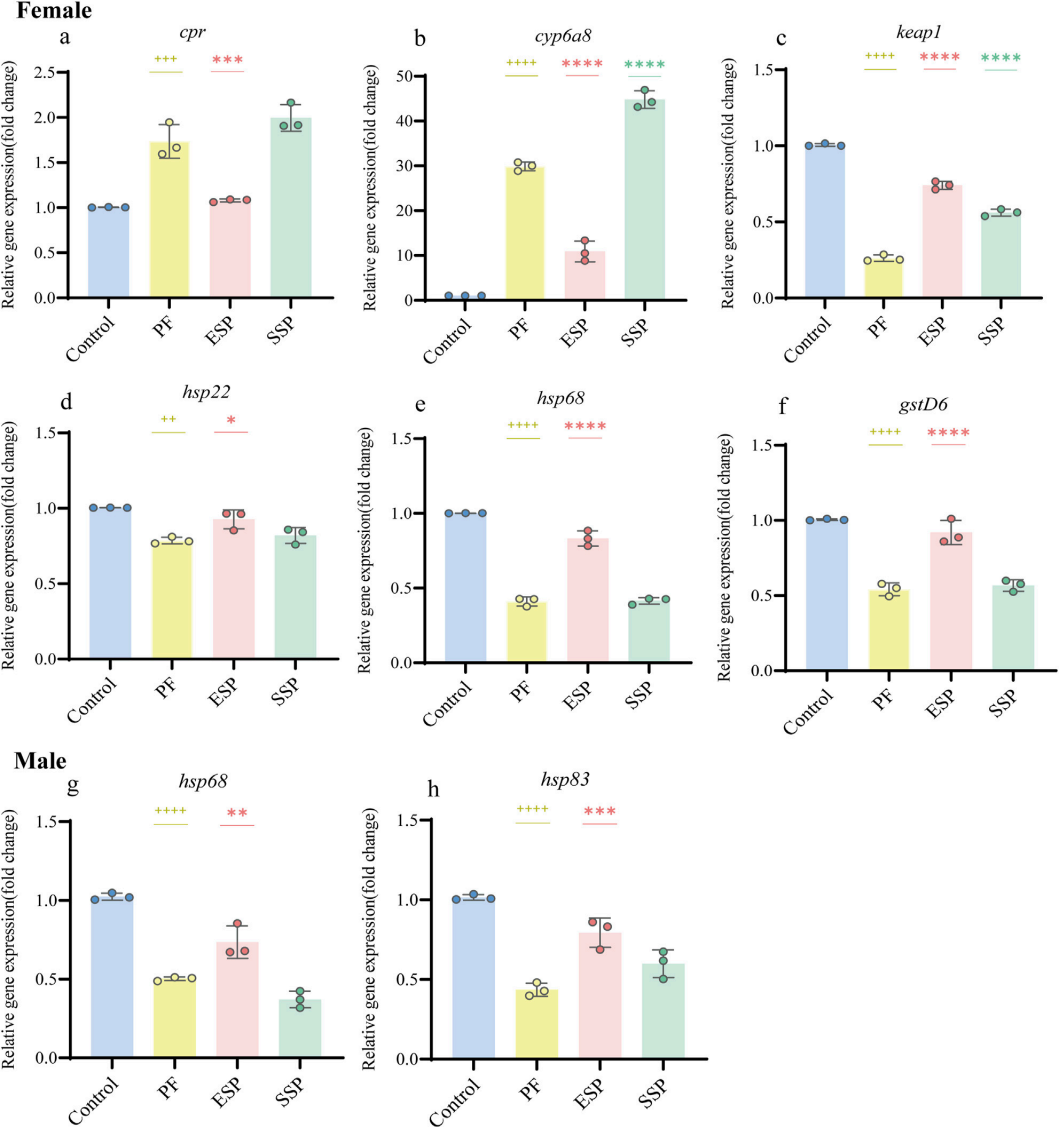
Stable gene expression in PF, ESP, SSP groups in female and male *Drosophila*. **(a–f)** represent gene expression of cpr, cyp6a8, keap1, hsp22, hspp68, gstD6 in female *Drosophila*. **(g,h)** represent gene expression of hsp68 and hsp83in male *Drosophila*. + indicates a significant difference compared to the Control, p < 0.05 (^++^p < 0.01,^+++^p < 0.001,^++++^p < 0.0001), *indicates a significant difference compared to the PF, p < 0.05 (**p < 0.01, ***p < 0.001, ****.

Analysis of upregulated and downregulated genes by qPCR: In the female group of PF, ESP and SSP, the overall expression of the *cpr* gene showed an upward trend, and ESP was a significantly different compared with PF (Figure 4A, p < 0.001). The overall expression of the *cyp6a8* gene showed an upward trend, and ESP and SSP were significantly different compared with PF (Figure 4B, p < 0.0001). The overall expression of the *keap1* gene showed a downward trend, and ESP and SSP were significant difference compared with PF (Figure 4C, p < 0.0001). The overall expression of the *hsp22* gene showed a downward trend, and ESP was a significant difference compared with PF (Figure 4D, p < 0.05). The overall expression of *hsp68* and *gstD6* genes showed a downward trend, and ESP was a significant difference compared with PF (Figure 4E, p < 0.0001; Figure 4F, p < 0.0001). In the male group, the overall expression of *hsp68* and *hsp83* genes showed a downward trend, and ESP a significant difference compared with PF (Figure 4G, p < 0.01; Figure 4H, p < 0.001).

Based on the above results. In the study of the detoxification combination of TCM, we can choose to detect the upregulation of *cpr* and *cyp6a8*, and the downregulation of *keap1*, *hsp22*, and *hsp68*, and *gstD6* in female fruit flies to judge the detoxification combination trend of Chinese medicine. In male fruit flies, we can detect the downregulation of *hsp68*, *hsp83* to judge.

### 3.4 Transcriptome sequencing to explore the potential mechanism

We performed a clustering analysis on 24 samples, and found that the majority (80%) of each group was classified into one category, indicating good biological repetition (Supplementary Material 4; Figure 6). A comparative analysis of male and female fruit flies revealed that a portion of genes showed contrasting patterns of expression, with upregulation in one sex corresponding to downregulation in the other. And we created a volcano plot and analyzed the up-and-down regulation (Supplementary Material 4; Figure 7).

**FIGURE 5:**
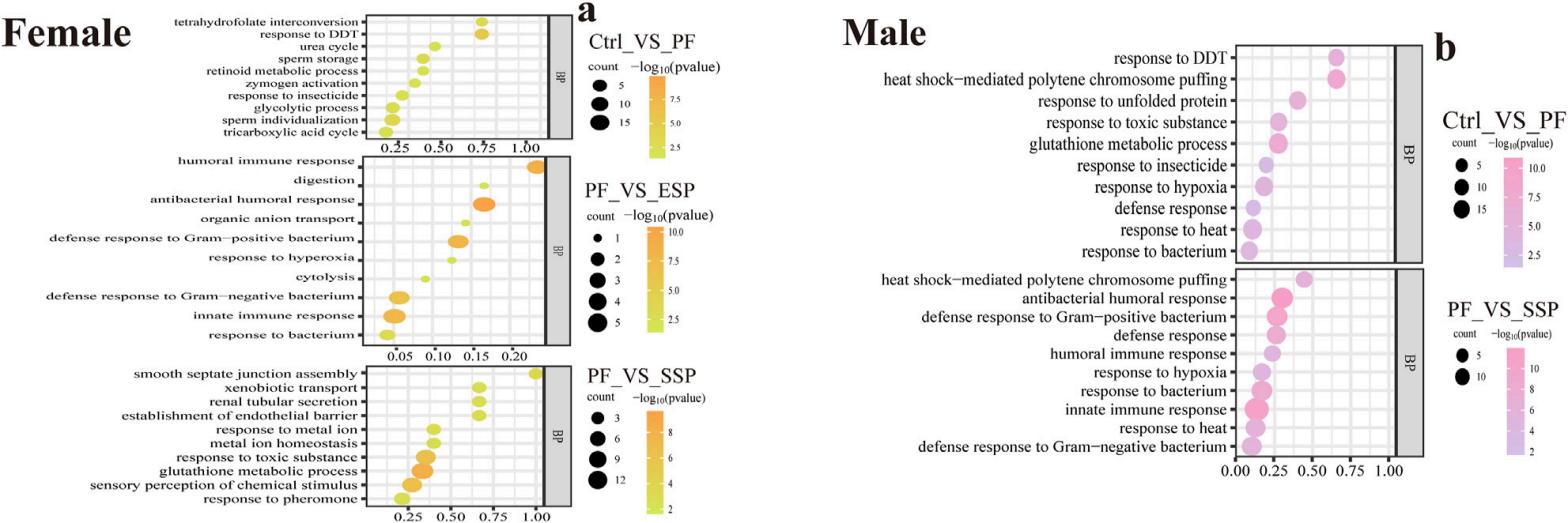
GO enrichment analysis, upregulated gene changes in biological processes. **(a,b)** PF exhibits upregulated expression compared to Control, ESP shows upregulated expression compared to PF, SSP demonstrates upregulated expression compared to PF in female and male *Drosophila*.

In GO enrichment, based on qPCR results, we further investigated the mechanisms of ESP, and SSP to fruit flies. In female fruit flies with upregulated gene expression, the PF group, compared to the Control group, primarily influenced biological processes such as response to DDT (Dichlorodiphenyltrichloroethane), response to insecticide, and the glycolytic process, involving processes related to DDT or insecticides and glycolysis. In the ESP group compared to the PF group, there was an increase in gene expression related to humoral immune response and innate immune response. Following the ESP and SSP groups, compared to the PF group, there was an increase in gene expression associated with innate immune response, response to toxic substances, glutathione metabolic process, and response to pheromone. Therefore, ESP and SSP can increase the fruit fly’s resistance to external adversities by upregulating the genes involved in these biological processes (Figure 5A).

In male fruit flies, the PF group, compared to the Control group, influenced biological processes such as response to DDT, heat shock-mediated polytene chromosome puffing, and glutathione metabolic process, similar to female fruit flies. In the SSP compared to the PF, there was an increase in gene expression related to innate immune response, response to heat, and humoral immune response. The upregulation of genes in these biological processes by SSP helps maintain homeostasis in the fruit flies (Figure 5B). In the female fruit flies with downregulated gene expression (Supplementary Material 4; Figure 8A).

**FIGURE 6:**
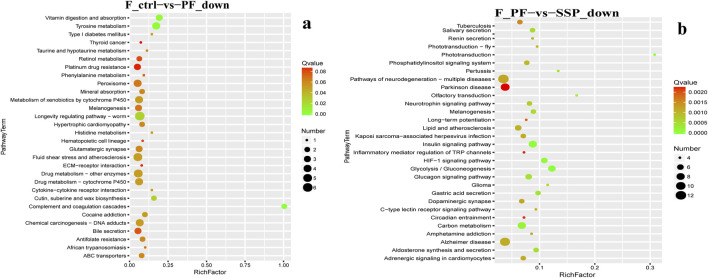
KEGG downregulated Gene Enrichment. **(a)** PF shows downregulated expression compared to Control. **(b)** SSP shows downregulated expression compared to PF. The horizontal axis represents RichFactor.

In KEGG enrichment, in female fruit flies with downregulated gene enrichment, the PF group, compared to the Control group, is mainly enriched in the Vitamin digestion and absorption and Tyrosine metabolism pathways (Figure 6A). The ESP group shows no significant enrichment and has fewer genes (Supplementary Material 5; Figure 1A). Compared to the PF group, the SSP group is mainly enriched in the Insulin signaling pathway and Carbon metabolism pathway (Figure 6B).

**FIGURE 7:**
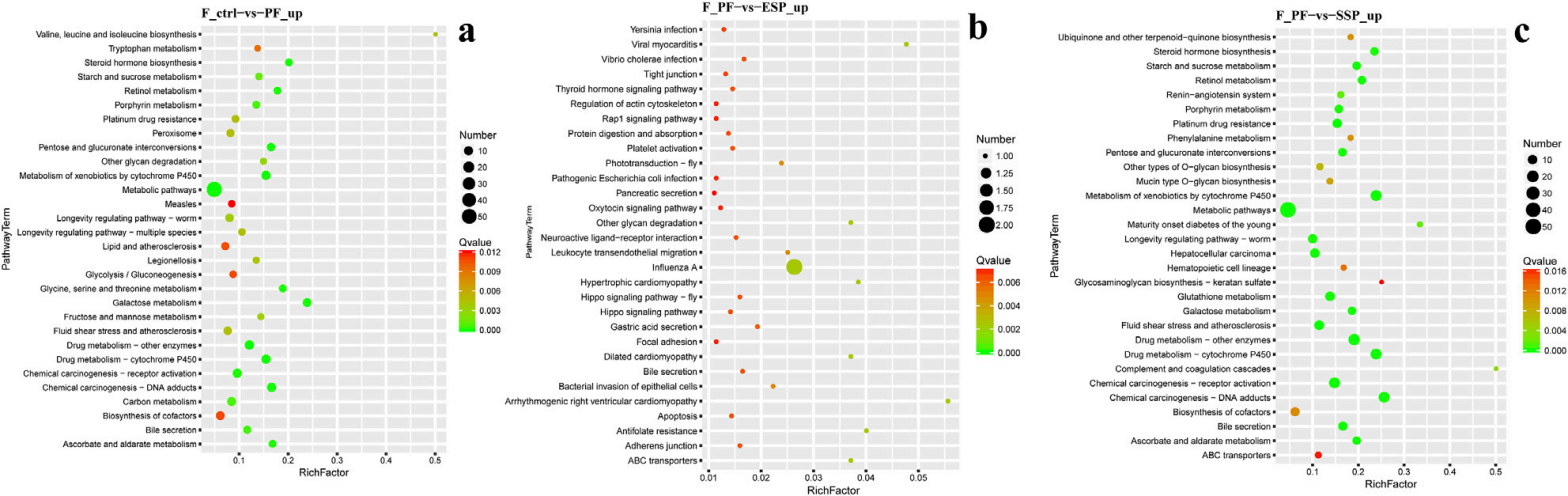
KEGG upregulated Gene Enrichmen **(a)** PF exhibits upregulated expression compared to Control. **(b)**. ESP shows upregulated expression compared to PF. **(c)** SSP demonstrates upregulated expression compared to PF.

In female fruit flies with upregulated gene enrichment, the PF group, compared to the Control group, is mainly enriched in Metabolic pathways and Galactose metabolism pathways (Figure 7A) The ESP group, compared to the PF group, is mainly enriched in the Influenza A pathway (Figure 7B). The SSP group, compared to the PF group, is mainly enriched in the Metabolism of xenobiotics by cytochrome P450 and Drug metabolism -cytochrome P450 pathways (Figure 7C). In addition, the KEGG enrichment analysis of males is provided in Supplementary Material 5.

### 3.5 Ershen Pills’ lowest toxicity in PF combinations and detoxification trend viaformulation in *Vibrio fischeri* toxicity assays

**FIGURE 8:**
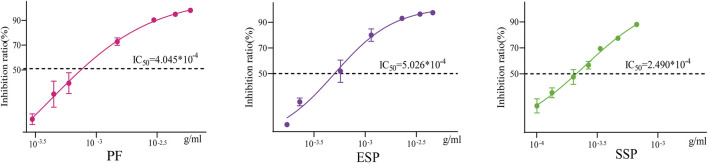
Inhibition rate-concentration curve The horizontal axis represents concentration, and the vertical axis represents the luminescence inhibition rate. The middle line in the curve represents the concentration corresponding to the IC50 value.

We tested the effect of TCM on *Vibrio fischeri* by detecting its bioluminescence intensity. We calculated the bioluminescent inhibition rate using the formula, and determined the concentration at which 50% inhibition (IC_50_) is achieved. The smaller the IC_50_, the less concentration of the drug is required to inhibit 50% of the bioluminescence of *Vibrio fischeri*, implying greater potential toxicity. The IC_50_ of PF is 4.045 × 10^−4^, the IC_50_ of ESP is 5.026 × 10^−4^, the IC_50_ of SSP is 2.490 × 10^−4^ (Figure 8). By comparing IC_50_, we found that ESP > PF, ESP > SSP, and PF > SSP. This suggests that when PF is combined to form ESP and SSP, ESP has the least potential toxicity to *Vibrio fischeri*, SSP has the most, and ESP shows a detoxification trend. This is also consistent with the survival analysis of fruit flies and enzyme detection trends.

### 3.6 Toxic component counts in PF, ESP, and SSP via similarity model screening (4, 16, 22)

We compared the MACCS molecular similarity of 1,277 toxic compounds of *Vibrio fischeri*, and screened out the compounds with a Tanimoto coefficient of ≥0.5. This resulted in 73,710 interaction points. These points were then imported into CytoNCA for Degree value analysis. We set the threshold for selection as half of the maximum Degree value of 180, which is 90. Eventually, we filtered out 304 compounds with a Degree value greater than 90 and used them as a toxic compound similarity dataset. For the acquisition of TCM ingredients, we obtained 102 components of PF from the literature (Koul et al., 2019). We also found 265 components of Fresh Ginger, 133 components of Jujube Dates, 64 components of Myristicae Semen in ESP, 176 components of Evodiae Fructus, 50 components of Schisandrae Chinensis Fructus in SSP from TCMSP (https://tcmsp-e.com). Each of these components was then matched with the previous 304 components for similarity. With a Tanimoto coefficient of ≥0.5 and the median of the maximum Degree value set as a threshold, we screened and found that PF had 4, ESP had 16, and SSP had 22 (Supplementary Material 6).

### 3.7 Toxic component counts in PF, ESP, and SSP after virtual screening (3, 5, 5)

Due to the use of docking software for force field treatment of molecular structures and batch molecular docking with proteins, even the same molecules from different TCMs docked with the same protein may not yield identical results. Therefore, we conducted clustering analysis on all docking results (Supplementary Material 7) to see if the same molecules from different TCMs cluster together when bound to proteins. LuxAB is a large complex, and it's not feasible to complete molecular docking with the protein in the docking process. By breaking down the protein structure into LuxA_A, LuxA_C, LuxB_B, and LuxB_D, molecules can fully compute and match docking with the protein, avoiding the local effects of molecular interaction with the protein and considering the diversity of molecular binding with the protein. Clustering analysis found that components with the same identification number are mostly classified into one category, indicating that the difference in binding of the same component from different TCMs to the same protein is not significant (Supplementary Material 7; Figure 1A).

**FIGURE 9:**
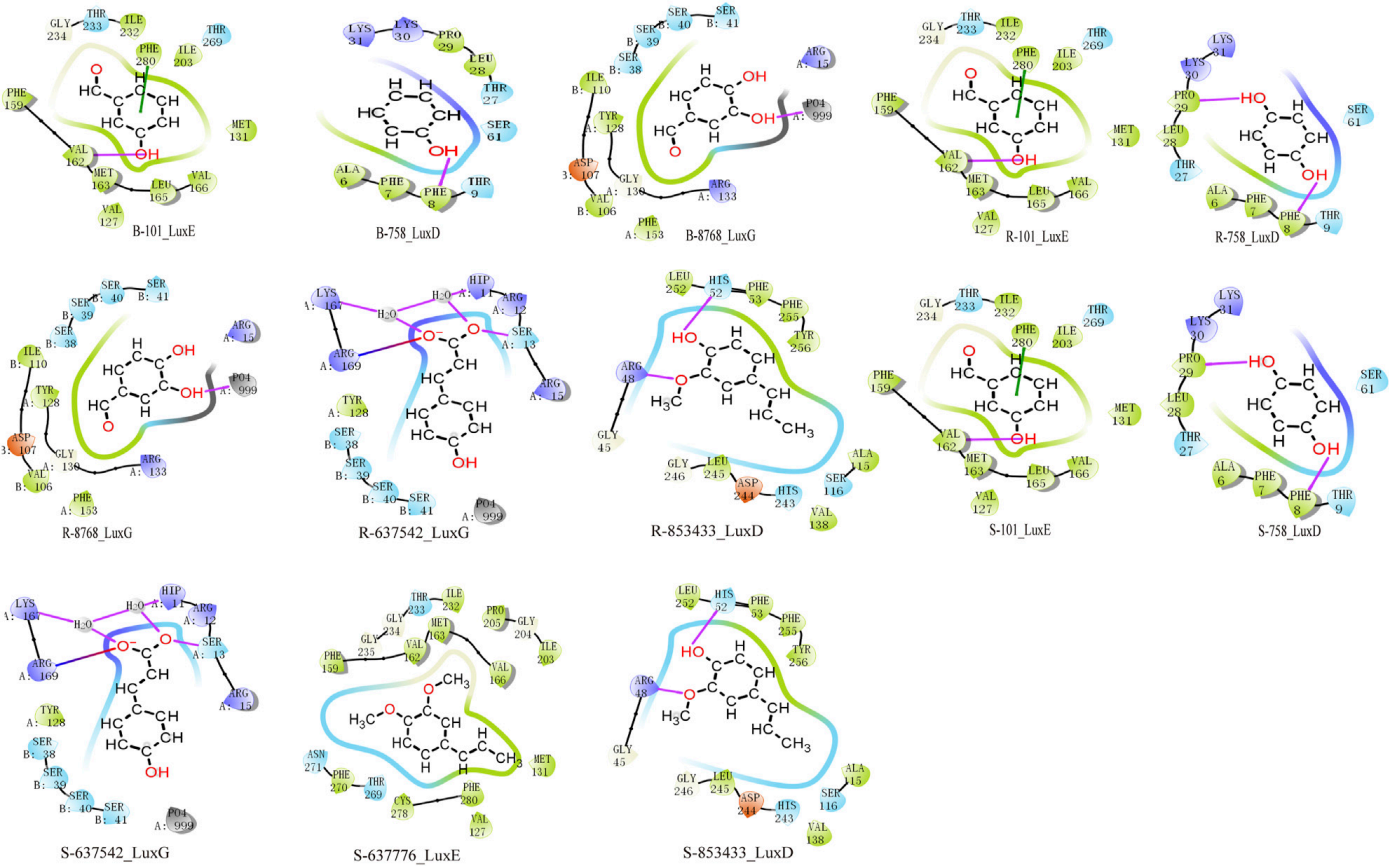
Molecular docking analysis in clustering includes components of Psoralea Fructus, Er Shen Pills, and Si Shen Pills with protein docking results. The red line represents the H-bond, the green line represents the Pi-Pi stacking and the line that is half blue and half red represents a salt bridge.

**FIGURE 10:**
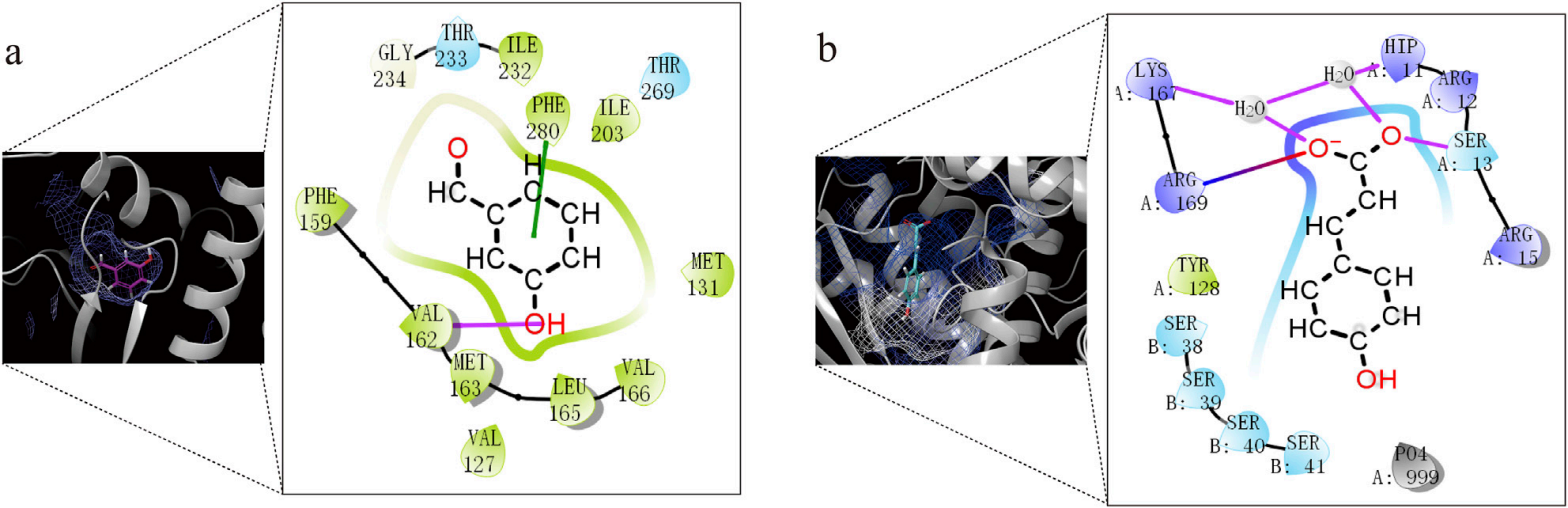
Docking analysis and visualization of the best-performing components in each TCM with protein binding pockets. **(a)** B-101_LuxE. **(b)** R-637542_LuxG.

Through analysis of the clustering diagram and docking results, we selected components for visualization and molecular interaction analysis with the protein based on the lowest docking score. The main binding method of molecules is hydrogen bond interaction. In Psoralea Fructus (PF), it mainly binds to the PHE of the Lux protein. Among them, B-101 has the largest number of bonds formed with LuxE. The hydroxyl group on B-101 forms a hydrogen bond with the VAL162 site of the LuxE protein, and its benzene ring forms a pi-pi stacking with the PHE280 site. The components R-101 and R-758 of Ershen Pills (ESP) bind to the PHE site of the Lux protein in the ways of pi-pi stacking and hydrogen bonding respectively; R-637542 and R-853433 mainly bind to the ARG site of the Lux protein through salt bridge and hydrogen bonds, respectively. The components S-101, S-758, R-637542 and R-85343 of Sishen Pills (SSP) mainly bind to the amino acid sites of the Lux protein, which is similar to that of ESP; S-637776 has no interaction bonds with LuxE (Figure 9). Due to the large number of dockings, based on the lowest docking score and the highest number of binding bonds, we selected the best ligand-receptor binding within each TCM for further analysis. In PF, B-101_LuxE was the strongest (−6.842), with binding sites at VAL162, PHE280 (Figure 10A). In ESP R-637542_LuxG (−7.487) was selected, with binding sites at LYS_A:167, ARG_A:169, HIP_A:11, SER_A:13 (Figure 10B). SSP was similar to ESP.

### 3.8 The stable interaction of B- 101 with LuxE and the stable interaction of R - 637542 with LuxG

**FIGURE 11:**
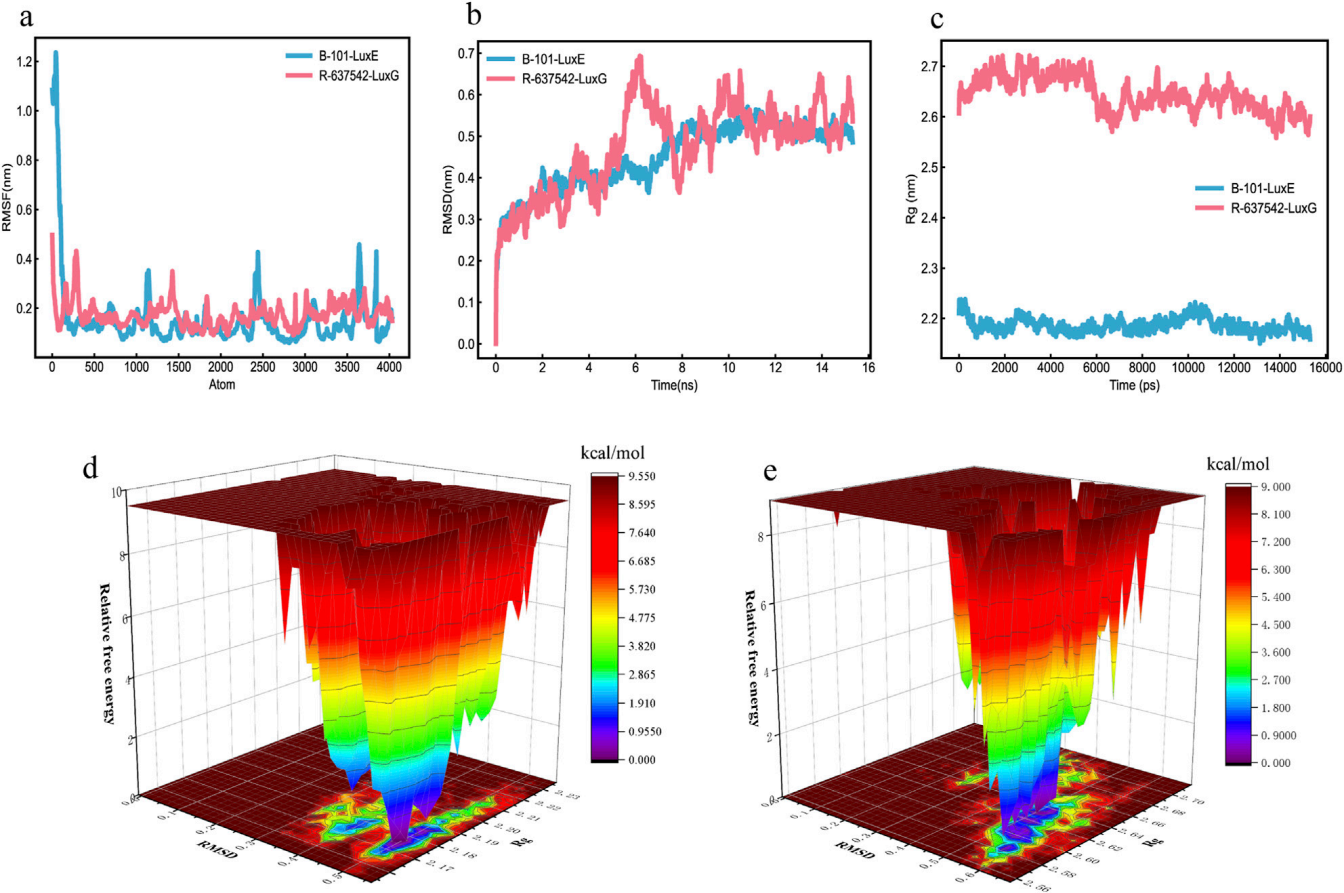
MD Molecular Dynamics Simulation Results **(a)** RMSD. **(b)** RMSF. **(c)** Rg. **(d)** Free energy landscape of B-101_LuxE. **(e)** Free energy landscape of R-637542_LuxG.

We use Schrödinger software for molecular docking of molecules and proteins, adopting a semi-flexible docking approach. However, receptors in a real environment are dynamically changing, so molecular dynamics simulation is used to evaluate the dynamic interactions between molecules and proteins. Therefore, to further verify the binding degree and stability of TCM components with proteins, molecular dynamics simulations of more than 10 ns were conducted. We selected the ligand-receptor complexes (B-101_LuxE, R-637542_LuxG) from the molecular docking results, which were screened for molecular dynamics simulation. RMSF is used to assess the fluctuation mobility of protein amino acid residues, with smaller values indicating less fluctuation. Between 100–4000 atoms, the RMSF values of B-101_LuxE, R-637542_LuxG, fluctuate within a range of 0.4 nm, indicating that the TCM components B-101, R-637542 have a minimal impact on the overall structure of the protein (Figure 11A; Feng et al., 2024).

RMSD is used to further assess the stability of the binding between molecules and proteins. A smoother RMSD curve indicates more stable binding between the molecule and the protein. Within 0–15 ns, the fluctuations of B-101_LuxE, R-637542_LuxG are within 0.5 nm, indicating stable binding between the molecules and proteins (Figure 11B). The protein folding state is further evaluated through Rg. As time increases, a larger Rg value indicates a process of protein unfolding, while a smaller value indicates folding (Figure 11C). We also analyzed the number of hydrogen bonds at the binding sites in the complexes. The number of hydrogen bonds for B-101_LuxE and R-637542_LuxG remains stable throughout the process (Supplementary Material 8).

We created free energy landscape maps using RMSD, Rg, and Gibbs relative free energy as the X, Y, and Z-axes, respectively, to plot the conformation with the lowest energy throughout the binding process of the simulated receptor and molecule. This evaluates the strength of the interaction between the ligand and the protein. If the interaction between the ligand and the protein is strong, the peaks of the free energy landscape map are distinct and smooth, with fewer clusters of minimal energy on a surface with multiple undulations. The free energy landscape maps of B-101_LuxE, and R-637542_LuxG show almost a single and smooth cluster of minimal energy (Figures 11D,E), indicating stable binding between the ligand and the receptor. In conclusion, based on the various indicators from the MD simulation analysis, the interactions of B-101 with LuxE, and R-637542 with LuxG are stable.

## 4 Discussion

### 4.1 Lifespan assays

The Kaplan-Meier method is a vital way to create a survival curve estimating events of interest (such as death or other factors) as follow-up time elapses (Park S. Y. et al., 2021). In our study, all the experimental subjects, fruit flies, died during the course of the experiment, with no instances of mid-term withdrawal or survival. Consequently, we are utilizing both the Mean and Median values to holistically evaluate the average survival time of the fruit flies (Park S. H. et al., 2021).

In our lifespan analysis, we have found that psoralen, a component in PF, acts as a pesticide and has lethal effects on insects (Britto et al., 2021). Simultaneously, we have identified that ESP and SSP groups have a 50% survival rate and lifespan greater than the PF group. This indicates that at such concentrations, ESP and SSP have pharmacological effects that can prolong the lifespan of female fruit flies. These phenomena are related to the toxicity reduction effect of PF when it is combined to form ESP and SSP, which is consistent with our hypothesized results. In addition, there was no significant difference between the PF group and the control group in the female group. This might be because female fruit flies developed resistance to PF, which prolonged the survival time of fruit flies in the PF group.

Based on the analysis of the survival duration and the median survival rate of female and male fruit flies, in male fruit flies, the ESP group conforms to the normal expectation, that is, since no toxic drugs were added to the control group, the median survival rate and survival time should be the longest. Therefore, male fruit flies may exhibit higher sensitivity to the combined herbal medicine and are better candidates for the survival analysis of the detoxification combined TCMs.

### 4.2 The trend of change on AchE, CarE and GST

Various external environmental factors can lead to the inhibition (Bianchini et al., 2022; Evelyn et al., 2024; Ogunsuyi et al., 2022; Oyeniran et al., 2021) or increase (Fournier et al., 1993; Hu et al., 2019) of *Drosophila* AChE activity. The occurrence of this inhibitory or inducing effect is dependent on the duration of exposure of fruit flies to various substances at different concentrations. If the exposure time is short, fruit flies can induce AChE activity, resulting in drug resistance against external influences (such as those containing organophosphorus pesticides). Consequently, if the exposure time is longer, the AChE activity in fruit flies might transition from induction to inhibition, causing AChE activity to fall below normal levels due to environmental influences.

In the experiment, the PF group inhibited AchE compared with the control group, while Ershen Pills (ESP) and Sishen Pills (SSP) could reduce this inhibition, which indicates that the toxic effects of ESP and SSP may be weaker than those of PF. In addition, PF only has an inhibitory effect on AChE activity in female fruit flies, but induces it in male fruit flies. This could be because the male fruit flies are still in a state of increased AChE activity, and both ESP and SSP also showed a detoxification trend.

The carboxylesterase is a multifunctional superfamily that is ubiquitous in all organisms. It plays a crucial role in detoxifying exogenous compounds (Yu et al., 2009). Carboxylesterase is also a type of metabolic enzyme relevant to pesticide resistance. It participates in an insect’s resistance to carbamate and pyrethroid pesticides through gene amplification, upregulation, and coding sequence mutations (Li et al., 2007). Previous research has found that over-expression of CarE-related genes and increased CarE activity were the primary mechanisms driving pesticide metabolic resistance (Feng et al., 2018; Li et al., 2021; Wang et al., 2015; Wang et al., 2016).

In contrast, in our experiment, we observed the inhibition of CarE activity rather than its overexpression. The reason for this might be that the effect has changed from induction to inhibition, thus reducing the CarE activity to below the normal level. This can also explain the inhibitory effect of PF on CarE activity, and the fact that ESP and SSP reduce this inhibitory effect, showing a detoxification trend.

Glutathione S-transferase (GST) is an important antioxidant enzyme involved in the Phase II detoxification system (Sau et al., 2010). It plays a pivotal role in the detoxification of both endogenous and exogenous compounds, and is also involved in intracellular transport, biological synthesis of hormones, and protection against oxidative stress. GST can metabolize pesticides by promoting their reductive dehydrochlorination or through a conjugal reaction with reduced glutathione. Additionally, it assists in removing toxic oxygen radicals produced by pesticide action (Enayati et al., 2005). Increases in GST activity can occur at different time points under various dosages and environmental conditions (Piccoli et al., 2019). Differences may exist between females and males, with potential instances where GST activity decreases in males but exhibits no impact on females (Dos Santos Moysés et al., 2017; Siddique et al., 2009). Previous research suggests that a reduction in GST activity in the black-bellied fruit fly exposed to tannery wastewater could be an indication of toxicity (Hayes and McLellan, 1999; Landi, 2000). An increase in GST activity after fruit flies were exposed to MeHg^+^ may serve as a measure of oxidative reduction status and inflammatory response (Piccoli et al., 2019). We propose that any substance causing a decrease or increase in GST activity in the black-bellied fruit fly could potentially serve as an indicator of toxicity. This depends on the conditions set for the fruit fly and the relevant assessments made.

In our experiment, compared with the control group, the PF group induced the activity of GST. However, ESP and SSP could reduce this induction and bring the GST activity to a level comparable to that of the control group. This further demonstrates the detoxification trend observed in ESP and SSP, which are formed through PF combination.

### 4.3 Quantitative real-time PCR

In the selection of PCR gene primers, we chose the reported primer sequences (Supplementary Material 2). The selected 23 genes are genes expressed by fruit flies under the influence of the external environment (such as pesticides, heavy metals, etc.), mainly involving oxidative stress, toxic metabolism, etc., (Daborn et al., 2007; Frat et al., 2021; Mombach et al., 2022; Willoughby et al., 2006).

Through the results of the Venn diagram, we found differences in the upregulated and downregulated genes expressed in males and females. The differences in PF, ESP, and SSP are mainly in the expression of upregulated genes. In further differential analysis, only *hsp68* showed a consistent trend of downregulated expression in both sexes. This difference in expression is related to the sex of the fruit fly and the dose of the drug (Wang et al., 2013).

### 4.4 GO enrichment analysis

In female fruit flies with downregulated gene expression, the PF group, compared to the Control group, primarily influenced biological processes involving biological defense mechanisms, environmental perception and response, and signal transduction, all of which regulate basic biological mechanisms. The changes in biological processes caused by ESP and SSP also relate to the biological processes induced by PF, but with significantly fewer downregulated genes. This suggests that after combination with ESP and SSP can reduce the downregulation of genes related to biological defense mechanisms, environmental perception and response, and signal transduction. This reduction in downregulation helps alleviate issues such as energy metabolism disorders, cell damage, and decreased immunity, thereby improving the health and survival of the fruit flies (Supplementary Material 4; Figure 8A).

In male fruit flies, the biological processes caused by PF are similar to those in females. However, ESP and SSP involve an increased number of genes downregulated related to biological defense mechanisms, signal transduction mechanisms, homeostasis maintenance capabilities, and basic biological characteristics for environmental adaptation. This indicates that in male fruit flies, ESP and SSP do not effectively demonstrate the effects of detoxification (Supplementary Material 4; Figure 8C).

In the enrichment of biological processes with upregulated genes, both female and male fruit flies exhibit the beneficial effects of the ESP and SSP groups on changes in enriched gene biological processes. By up-regulating genes related to innate immune response and defense response, they maintain the homeostasis of the fruit fly organism, demonstrating the attenuating combination effects of ESP and SSP. A summary was also made for Cellular Component (CC), Molecular Function (MF), and Biological Process (BP) (Supplementary Material 4; Figure 8D).

In summary, in both female and male fruit flies the PF groups mainly influence the upregulation of genes involved in biological processes such as response to DDT and heat shock-mediated polytene chromosome puffing. After combination, ESP and SSP primarily improve the fruit flies’ condition by up-regulating genes involved in biological processes such as innate immune response and defense response.

### 4.5 KEGG pathway enrichment analysis

In female fruit flies, among the genes enriched in downregulated pathways, the insulin signaling pathway is a key cellular signaling pathway that plays a significant role in the regulation of carbohydrate, fat, and protein metabolism. The carbon metabolism pathway is crucial for energy production and the synthesis of organic molecules within organisms (Biglou et al., 2021; Mattila and Hietakangas, 2017). SSP primarily affects the metabolism of sugar, fat, and protein and the energy balance in *Drosophila* by down-regulating genes in the Insulin signaling pathway and Carbon metabolism pathway. In male fruit flies, ESP may affect metabolic regulation, cell differentiation, and signal transduction during development by downregulating genes in the PPAR signaling pathway and Lysosome pathway (Yazar et al., 2021; Zipper et al., 2020) and the degradation and recycling of cellular materials, cell death, and the autophagy process (Jacomin et al., 2016; Rigon et al., 2021). SSP may regulate lifespan, immune response to parasitic infections, and bacterial infections by downregulating genes in the Toxoplasmosis, Spliceosome, and Protein processing in endoplasmic reticulum pathways (Carter, 2013; Ogienko et al., 2022; Sun et al., 2013).

In female fruit flies enriched with upregulated genes, those in the Influenza A pathway related to the transmission and infection mechanisms of Influenza A can increase sensitivity to the virus. ESP can exert antiviral effects by upregulating genes in the Influenza A pathway (Mongelli et al., 2022; Smelkinson et al., 2017). However, it is not very relevant to the study of TCM combinations. SSP mainly regulates the metabolism of exogenous compounds, drugs, and toxins by upregulating genes in the pathways of Metabolism of xenobiotics by cytochrome P450 and Drug metabolism-cytochrome P450 (Chung et al., 2009; Giraudo et al., 2010). In male fruit flies, ESP mainly affects health and development by up-regulating genes in pathways such as Thiamine metabolism and Folate biosynthesis (Blatch et al., 2010; Sannino et al., 2018). SSP mainly regulates protein digestion and absorption in *Drosophila* by up-regulating genes in pathways such as Protein digestion and absorption and DNA replication, thereby modulating nutrient intake and metabolism (Chopra et al., 2022; Holtof et al., 2019; Lemaître et al., 2013) and maintaining the stability of the *Drosophila* genome and genetic information (Hua and Orr-Weaver, 2017; Sibon et al., 1997).

In conclusion, ESP and SSP mainly regulate the health of *Drosophila* by downregulating genes related to the insulin signaling pathway and so on, and upregulating genes related to the pathways of Metabolism of xenobiotics by cytochrome P450 and Drug metabolism-cytochrome P450.etc.

### 4.6 *Vibrio fischeri* toxicity assays

The *Vibrio fischeri* bioluminescence inhibition assay uses a rapid, sensitive, and cost-effective method to evaluate bio-effects, which can display specific information about toxicity or ecotoxicity (Abbas et al., 2018; Parvez et al., 2006). Various factors can affect the detection. In cases where the colour or turbidity of the water sample is high, there may be a light loss caused by light absorption or scattering (Standardization, 2007). and samples with a pH value out of the range of 6.0∼8.5 can affect the bioluminescence of bacteria (Postma et al., 2002). During the detection, surprisingly, SSP did not show a detoxification trend in the acute toxicity tests, but we found that the IC_50_ of SSP was very close to that of PF. This indicates that SSP may also show a detoxification trend after the combination. This could be influenced by its brown colour, or it could be due to the possibility that multiple components in SSP interact with *Vibrio fischeri* greatly inhibiting the bacteria’s bioluminescence signal. Between the two, the latter is more likely. This further suggests that not all traditional Chinese medicinal materials are suitable for toxicity assessment using the *Vibrio fischeri* bioassay.

### 4.7 Toxic components screening

We have found that there are some problems in the model building based on conventional QSAR (Sabando et al., 2022; Sigurnjak Bureš et al., 2021; Wunsch et al., 2021), such as not being able to include all data when constructing the training and test sets. There are also discrepancies in the evaluation and selection of the model after construction. These issues may lead to the loss of some data. Moreover, building the training and test sets, and the evaluation screening after model construction are time-consuming, which greatly reduces the efficiency of drug compound screening. We rely on the established structure similarity of toxic ingredients, directly matching the screened ingredients with these toxic ones, which reduces the data loss in model construction and data transformation. A highlight of this approach is its speed, high operability, and ability to use various similarities between compound structures. However, there are also some issues. For example, when performing similarity matching between toxic ingredients and using high similarity as model construction standards, it may filter out some ingredients that do not have high similarity but display considerable toxicity. This is a drawback. Additionally, this situation may exist in the filtering threshold set for Degree value. In response to this, we can only ensure the uniformity of data model construction conditions, to minimize data loss, and improve screening efficiency and quality as much as possible.

The sequential application of similarity model-based toxic component screening followed by molecular docking validation was implemented because these two methodologies employ distinct screening criteria: the former utilizes a toxicity dataset derived from *Vibrio fischeri* bioassays, while the latter is grounded in the luminescence inhibition mechanism of *Vibrio fischeri*. Their combined application significantly enhances the precision of toxicant identification.

### 4.8 Docking analysis

In PF, B-101 (3-hydroxybenzaldehyde) forms a hydrogen bond with VAL162 through its hydroxyl group, and its phenyl ring forms a Pi-Pi stacking with the phenyl ring of PHE280. In ESP, R-637542 ((E)-3-(4-hydroxyphenyl)prop-2-enoic acid) forms a hydrogen bond with LYS_A:167 and H_2_O, which in turn binds to another amino acid residue, HIP_A:11, and the ester oxygen anion of R-637542. ARG_A:169 forms a salt bridge with the ester oxygen anion of R-637542, and the oxygen of the carbonyl group in the ester of R-637542 is bound by hydrogen bonds with H_2_O and SER_A:13. In SSP, S-63776 does not form any interaction bonds with LuxE. It is possible that the two ether bonds in S-63776 have relatively low reactivity, making it difficult for them to interact with the LuxE protein. The binding of these TCM components to protein residues is a key mechanism in inhibiting the luminescence of *Vibrio fischeri*.

In our docking studies, we have found that having the lowest docking score does not necessarily prove that the binding between the ligand and the receptor is favourable, nor does it mean that there are more bonds formed between the molecule and the protein. A comprehensive evaluation based on the types and quantities of the bonds formed is also necessary (Guedes et al., 2014; Kim and Skolnick, 2008). We have comprehensively judged and selected these components for binding with the protein complex. Through the analysis of the above docking results (Figure 9), we found that compounds with hydroxyl and ester groups on the benzene ring form more binding bonds with Lux protein and have lower binding energy. From this, it can be inferred that phenolic and aromatic ester compounds have a strong interaction with *Vibrio Fischeri*.

### 4.9 Molecular dynamics simulations analysis

The duration of molecular dynamics simulations can also affect the assessment of ligand-receptor binding, such as using a 10 ns simulation to evaluate the folding and structural stability of proteins at a constant PH (Jansen et al., 2024). Additionally, 100 ns simulations have been used to study the intramolecular conformational changes within proteins (Koshy et al., 2010), and different simulation durations have been applied to various modules within MD simulations (Janowski et al., 2016). Moreover, while there are some differences among the force fields used during the simulations, these differences tend to decrease with sufficiently long simulations (Janowski et al., 2016). Based on the aforementioned studies, selecting simulation durations greater than 10 ns allows for the assessment of molecular binding stability with proteins and the folding states of proteins. Finally, the interactions we obtained between B-101 and LuxE, as well as between R-637542 and LuxG, are stable. However, specifically, techniques such as SPR (Surface Plasmon Resonance) or ITC (Isothermal Titration Calorimetry) can be used to illustrate their direct binding affinities.

### 4.10 Supports and potential limitations in translational relevance

Supports: in *Drosophila* in terms of the conservation of genes and signaling pathways, *Drosophila* shares approximately 75% of the disease-causing genes with humans (Reiter et al., 2001), and cancer-related signaling pathways such as Ras, patched/hedgehog, and JAK/STAT are highly conserved during evolution (Harrison et al., 1995; Oro et al., 1997; Wassarman et al., 1995). Second, regarding the reproducibility of toxicological endpoints, the glutamine analog (Acivicin) and JAK inhibitor (methotrexate) discovered in drug screening using *Drosophila* have had their anti-tumor activities verified through mammalian models (Gremese et al., 2019; Yadav et al., 2016). In addition, in terms of the similarity of toxicological responses, studies have found that *Drosophila’s* responses to some neurotoxins are similar to the pathological processes of human nervous system diseases. For example, there are similarities to the neurodegeneration associated with Parkinson’s disease (Nitta and Sugie, 2022).

Potential limitations: Firstly, *Drosophila* has the homology and limitations of metabolic organs, the dominance of innate immunity, and the absence of adaptive immunity, etc., (Chatterjee and Perrimon, 2021; Hoffmann and Reichhart, 2002). Moreover, due to the disparity in life cycles, it is challenging to mimic the long - term toxicity accumulation associated with human chronic conditions like neurodegenerative diseases (Rubin and Lewis, 2000). Additionally, in terms of interspecies sensitivity, *Drosophila* exhibits a higher tolerance to certain insecticides, such as organophosphates, than mammals do (Atkinson, 2009).

## 5 Conclusion

Integrating *Drosophila* drug administration with *Vibrio fischeri* bioluminescence inhibition assays enables rapid toxicity trend evaluation of TCM combinations. Survival time analysis revealed detoxification trends in both ESP and SSP formulations, with male flies showing optimal responsiveness. Complementary enzymatic activity profiling further confirmed these detoxification patterns, demonstrating enhanced sensitivity when using female flies, thereby establishing sex-specific differential responses in toxicity assessment paradigms. qPCR quantification of detoxification-related genes (cpr↑, cyp6a8↑, keap1↓, hsp22↓, hsp68↓, gstD6↓, hsp83↓) revealed distinct toxicity modulation patterns in PF, ESP, and SSP formulations. Complementary validation through *Vibrio fischeri* bioluminescence inhibition assays demonstrated ESP’s superior detoxification capacity (IC_50_ hierarchy: ESP > PF > SSP), indicating that structural modification via PF formulation into ESP effectively attenuates compound toxicity. In further mechanism exploration, ESP and SSP can maintain the homeostasis of fruit flies by upregulating genes of biological processes such as innate immune response, defense response. They can also increase the fruit flies’ ability to cope with external environments by upregulating genes in pathways such as Metabolism of xenobiotics by cytochrome P450, drug metabolism − cytochrome P450, Protein digestion and absorption, and DNA replication. In the toxicity screening of *Vibrio fischeri*, the toxic components identified are 4 for PF, 16 for ESP, and 22 for SSP. PF, ESP, and SSP mainly target LuxD, LuxE, and LuxG. Further evaluation shows that B-101 has a stable effect on LuxE, with R-637542 having a relatively stable effect on LuxG.

## Data Availability

The data presented in the study are deposited in the NCBI repository, accession number PRJNA1275564.

## Abbreviations

PF, Psoraleae Fructus; ESP, Er Shen Pills; SSP, Si Shen Pills; AchE, Acetylcholinesterase; CarE, Carboxylesterase; FPKM, Fragments Per Kilobase of transcript per Million mapped reads; GST, Glutathione S-transferase; IC50, 50% inhibition; K- M curve, Kaplan-Meier curve; L- MD, Molecular dynamics; M- NPT, Number of particles, Pressure, Temperature; N- NVT, Number of particles, Volume, Temperature; Rg, gyration radius; RMSD, Root Mean Square Deviation; RMSF, Root Mean Square Fluctuation; TCM, Traditional Chinese Medicine; MACCS, Molecular ACCess System.
