# Supplementary material for: Integrating Drosophila and Vibrio fischeri models for toxicity evaluation: uncovering detoxification trends in psoralea Fructus-TCM formulations
Source: Front Pharmacol. 2025 Jun 13;16:1590929. doi: 10.3389/fphar.2025.1590929 (PMC12203615; doi:10.3389/fphar.2025.1590929)
Supplement: Supplementary file 1 [file Supplementaryfile1.docx]

**Supplementary documents**

**Supplementary 1**(Material and Methods)

All TCM were purchased from Beijing TRT Co., Ltd. (Beijing, China).

production lot number:

Fresh Ginger:2022070311

Ginseng:2022060904

Jujube Dates:2022060712

Psoraleae Fructus:2022060706

[Myristicae Semen](https://old.tcmsp-e.com/tcmspsearch.php?qr=Myristicae Semen&qsr=herb_en_name&token=7e95129e444f8614ae9dca160db85b74):2022061008

[Schisandrae Chinensis Fructus](https://old.tcmsp-e.com/tcmspsearch.php?qr=Schisandrae Chinensis Fructus&qsr=herb_en_name&token=7e95129e444f8614ae9dca160db85b74):2022060710

| Ershen Pills | Proportion |
| --- | --- |
| Psoraleae Fructus(dried ripe fruit;processed) | 2 |
| [Myristicae Semen](https://old.tcmsp-e.com/tcmspsearch.php?qr=Myristicae Semen&qsr=herb_en_name&token=7e95129e444f8614ae9dca160db85b74)(dried ripe fruit;processed) | 1 |
| Jujube Dates(dried ripe fruit;processed) | 1 |
| Fresh Ginger(fresh root;processed) | 1 |

| Sishen Pills | Proportion |
| --- | --- |
| Psoraleae Fructus(dried ripe fruit;processed) | 4 |
| [Myristicae Semen](https://old.tcmsp-e.com/tcmspsearch.php?qr=Myristicae Semen&qsr=herb_en_name&token=7e95129e444f8614ae9dca160db85b74)(dried ripe fruit;processed) | 2 |
| Jujube Dates(dried ripe fruit) | 2 |
| Fresh Ginger(fresh root) | 2 |
| Evodiae Fructus(dried ripe fruits;processed) | 1 |
| [Schisandrae Chinensis Fructus](https://old.tcmsp-e.com/tcmspsearch.php?qr=Schisandrae Chinensis Fructus&qsr=herb_en_name&token=7e95129e444f8614ae9dca160db85b74)(dried ripe fruit；processed) | 2 |

Supply 1(Material and Methods)

| TCM | Extraction yield(%) |
| --- | --- |
| Psoraleae Fructus | 16.95 |
| Ershen Pills | 17.44 |
| Sishen Pills | 23 |

**Supplementary 2**

q-PCR Protocol:

RNA is extracted as follows. Firstly Precool chloroform([C166260500](https://www.reagent.com.cn/goodsDetail/Chloroform-d,-for-NMR,-99.8-atom-%-D,-contains-1-v/v%-TMS/%E6%B0%AF%E4%BB%BF-d, 99.8 atom % D, %E5%90%AB1 v/v% TMS/9ce1c659ffb44704838cdd83eef64966" \t "https://www.reagent.com.cn/ProductSearch/_blank)，Sinopharm Chemical ReagentCo., Ltd) and isopropanol([40064360](https://www.reagent.com.cn/goodsDetail/Isopropanol/%E5%BC%82%E4%B8%99%E9%86%87/af1fe24d37704d9486bbf02a4696b7bd)，Sinopharm Chemical ReagentCo., Ltd) and 75% alcohol([80176961](https://www.reagent.com.cn/goodsDetail/Ethanol-75%/%E4%B9%99%E9%86%8775%/165bdf35dc51483196906957f655cd48)，Sinopharm Chemical ReagentCo., Ltd). Take 50-100 mg fly samples into 1.5 ml Eppendorf tube(EP) with 1 ml TRlzol([69131480](https://www.reagent.com.cn/goodsDetail/TRIZOL%EE%97%A5LS%EE%97%A5Reagent/80a755a2e9a8420b8d4cc659c6878293)，Sinopharm Chemical ReagentCo., Ltd) and homogenize on ice for 15 minutes until fully mixed. At 25 ℃ lysis for 10 minutes, add chloroform, vortex for 3 minutes, then centrifuge at 4℃, 12000 rpm, for 15 minutes. Take the upper colorless water phase(400ul), add an equal volume of isopropanol, shake up and down 4-5 times, let it stand at 25 ℃ for 10 minutes, then centrifuge at 4℃, 12000 rpm for 15 minutes. Discard the supernatant and add 75% ethanol, then centrifuge at 4℃, 7500 rpm for 5 minutes. Discard the supernatant, dry at 25℃ for 10 minutes, dissolve thoroughly in nuclease-free water(G4700-500ML, Servicebio Technology CO.,LTD, Wuhan) , and store at -80℃. RNA concentration determination by NanoDrop™ 8000 (ND-8000-GL, Thermo Fisher Scientific Inc.). cDNA Synthesis recording to *TransScript*^®^ First-Strand cDNA Synthesis SuperMix(AT311, TransGen Biotech Co., Ltd, Beijing) and qPCR quantifying gene expression by *PerfectStart*^®^ Green qPCR SuperMix(AQ601, TransGen Biotech Co., Ltd, Beijing) were achieved.


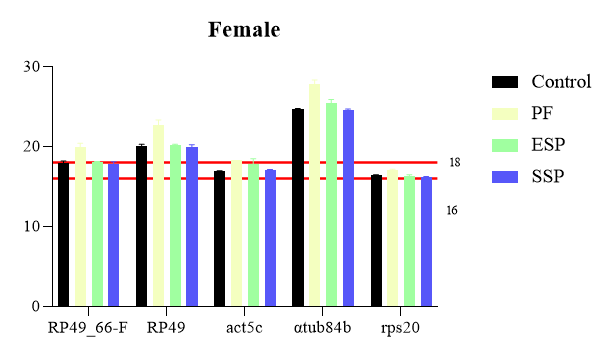


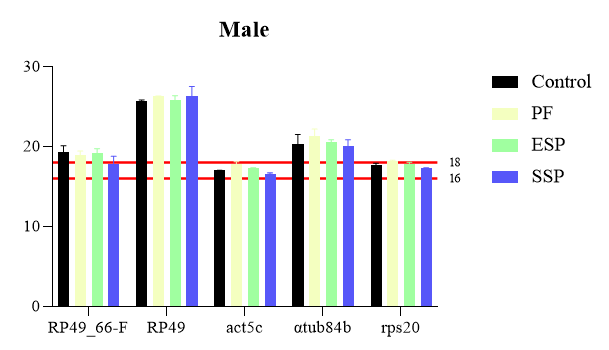


| RP49_66-F | CCAGTCGGATCGATATGCTAA |
| --- | --- |
| RP49_66-R | GTTCGATCCGTAACCGATGT |
| rp49-f | GCTAAGCTGTCGCACAAA |
| rp49-r | TCCGGTGGGCAGCATGTG |
| act5c-F | CACCGTCGACCATGAAGATCAAGA |
| act5c-R | AAGCACTTGCGGTGCACAATG |
| α-tub84b-F | TGCCCTTAACGTGGATCTGACTGA |
| α-tub84b-R | TGAATGGTGCGCTTGGTCTTGA |
| rps20-F | CACCCAAGGATATTGAGAAGCCC |
| rps20-R | TGGATTCTCATCTGGAAGCGATC |
| gstd6-F | TGCCTTCGATCTTCTCAACA |
| gstd6-R | CTGCCACTGAAAGCTGATTG |
| hsp70-F | AGCCGTGCCAGGTTTG |
| hsp70-R | CGTTCGCCCTCATACA |
| hsp40-F | GAGATCATCAAGCCCACCACAAC |
| hsp40-R | CGGGAAACTTAATGTCGAAGGAGAC |
| hsp23-F | GGTGCCCTTCTATGAGCCCTACTAC |
| hsp23-R | CCATCCTTTCCGATTTTCGACAC |
| cyp18a1-F | CGCTGAACGGATATGGCATTATC |
| cyp18a1-R | CTCATCATCAGGCTGCAGATCA |
| sod1-F | TGCGTAATTAACGGCGATGCCA |
| sod1-R | AGTCGGTGATGTTGACCTTGGTG |
| sod2-F | TTTCGCAAACTGCAAGCCTGG |
| sod2-R | TCTTCAGATCATCGCTGGGC |
| cat-F | CAACCCCTTCGATGTCACCA |
| cat-R | TCTGCTCCACCTCAGCAAAG |
| cyp6a2(F) | AAACGGTGCTGGAGGAAC |
| cyp6a2(R) | TTATGACCTGTGTGCCCTTC |
| cyp6a8(F) | GGCTGAGGTGGAGGAGGT |
| cyp6a8(R) | CGATGACGAAGTTTGGATGA |
| gstD2(F) | TGTCCACTGTCTCCACGTTC |
| gstD2(R) | GGAGTCACCTTCTTGGCATT |
| gstD7(F) | TGGCTGATATCGTCATCCTG |
| gstD7(R) | GCATTCTTAAGCCACCTCTCC |
| cyp6g1(F) | GCTGAAGGACGAGGCTGTGGT |
| cyp6g1(R) | CGGGCGTATCGGTTGTGGA |
| hsp22-F | GCCTCTCCTCGCCCTTTCAC |
| hsp22-R | TCCTCGGTAGCGCCACACTC |
| hsp68-F | GAAGGCACTCAAGGACGCTAAAATG |
| hsp68-R | CTGAACCTTGGGAATACGAGTG |
| hsp83-F | GGACAAGGATGCCAAGAAGAAGAAG |
| hsp83-R | CAGTCGTTGGTCAGGGATTTGTAG |
| cpr-F | TCCCGAAGGAAAGGAGAAAT |
| p38-F | ACGTTCCTACATCCAGTCACTTC |
| p38-R | CTTCCATTTGTCTACGGGCAAAT |
| cpr-R | AGAACGGCTGTAACGTGGAC |
| hsf-F | AGTGTGGAGGACTTGCTGCT |
| hsf-R | AAGCACATTCTGCTCCTCGT |
| phgpx-F | GCTGACGGATCTAAAGGAGAAGT |
| phgpx-R | GAAGTTCCACTTGATTCCGCTG |
| keap1-F | GGTGTTGCTGCAATTAACCAGTA |
| keap1-R | CTGTTGTCCACGTATTTGTTCGT |
| cnc-F | CTATACAGTACGACCATCGCCAAGGA |
| cnc-R | GATGTCCGGCACTGAAATGGGTATGT |
| sirt1-F | CACCTTAAGTTCGATGTGGAGCT |
| sirt1-R | TAATAGACTGCGTATCCGTGTCC |

Cite:

1.Molecular and biological effects of Cisplatin in Drosophila

2.Single and mixed exposure to cadmium and mercury in Drosophila melanogaster: Molecular responses and impact on post-embryonic development

3.Evaluating the insecticide resistance potential of eight Drosophila melanogaster cytochrome P450 genes by transgenic over-expression

4.A comparison of Drosophila melanogaster detoxification gene induction responses for six insecticides, caffeine and phenobarbital

**Supplementary 3**

**On heatmap clustering analysis of up-regulated and down- regulated gene expression. As following below:**

It was found that in female fruit flies (Fig. 1a),different groups were compared. In the Control group, *gstD6*, *keap1*, *cat*, *hsp68* showed high expression, while *cyp6a2*, *cpr*, *gstD2* showed low expression. In the PF group, *cyp6a2*, *cyp6a8*, *hsp83* showed high expression, *keap1*, *hsp22*, *hsp68* showed low expression. In the ESP group, *sirt1*, *cat* showed high expression, *gstD2*, *cpr* showed low expression. In the SSP group, *cyp6a2*, *cyp6a8*, *cyp6g1* showed high expression, *hsp83*, hsf showed low expression.

In male fruit flies (Fig. 1b), different groups were compared. In the Control group, *hsp83*, *hsp68* showed high expression, *gstD7*, *gstD2* showed low expression. In the EF group, *p38* showed high expression, *phgpx* showed low expression. In the PF group, there were no obvious highly expressed genes overall, *sirt1*, *hsf,* *hsp83*, *hsp68* showed low expression. In the ESP group, *sirt1*, *hsf*, *keap1*, *cnc*, *gstD7* showed high expression, cat, *hsp22* showed low expression. In the SSP group, *gstD2*, *gstD7*, *phgpx*, *sirt1* showed high expression, *hsp83*, *hsp68*, *hsp22* showed low expression.


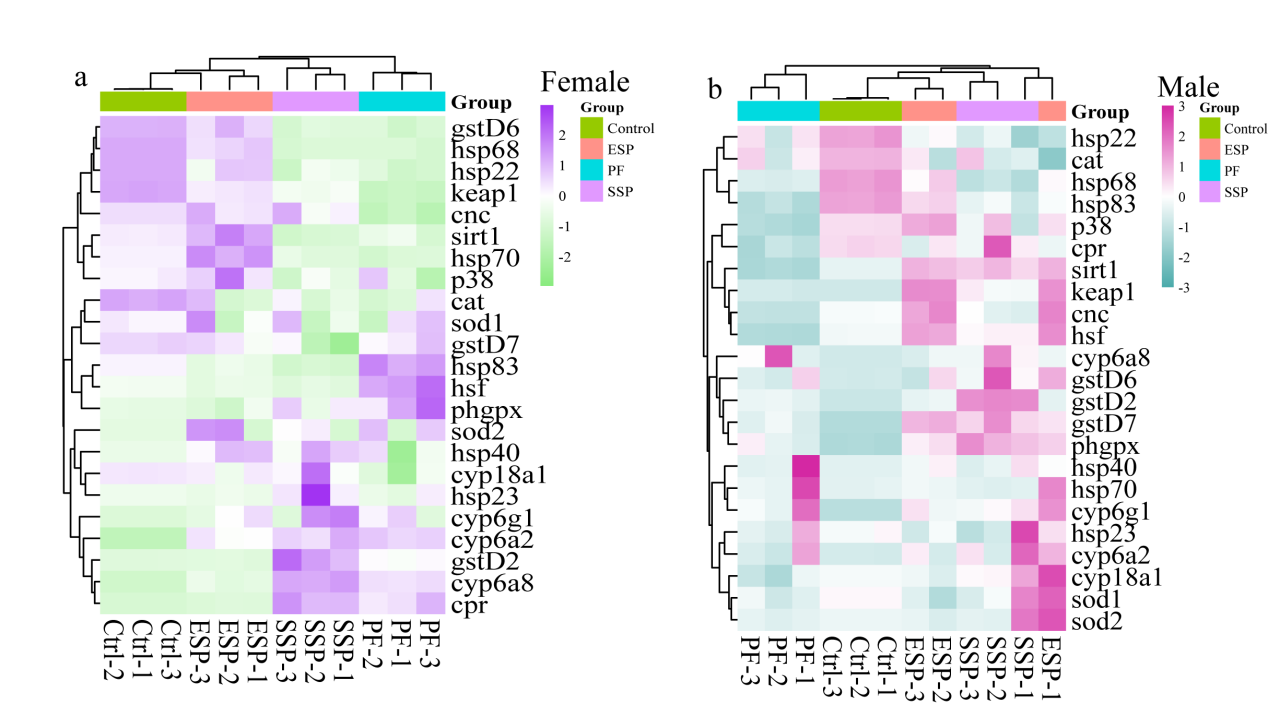


Fig . 1 TCM Effects on Gene Expression Heatmap Clustering in Male and Female Drosophila a, b Control, PF, ESP, SSP represent gene expression in three biological replicate samples. For females, the darker the purple, the higher the up-regulated gene expression; the darker the green, the lower the down-regulated gene expression. For males, the darker the red, the higher the up-regulated gene expression; the darker the gray-blue, the lower the down-regulated gene expression.

**Supplementary 4**

**clustering analysis**


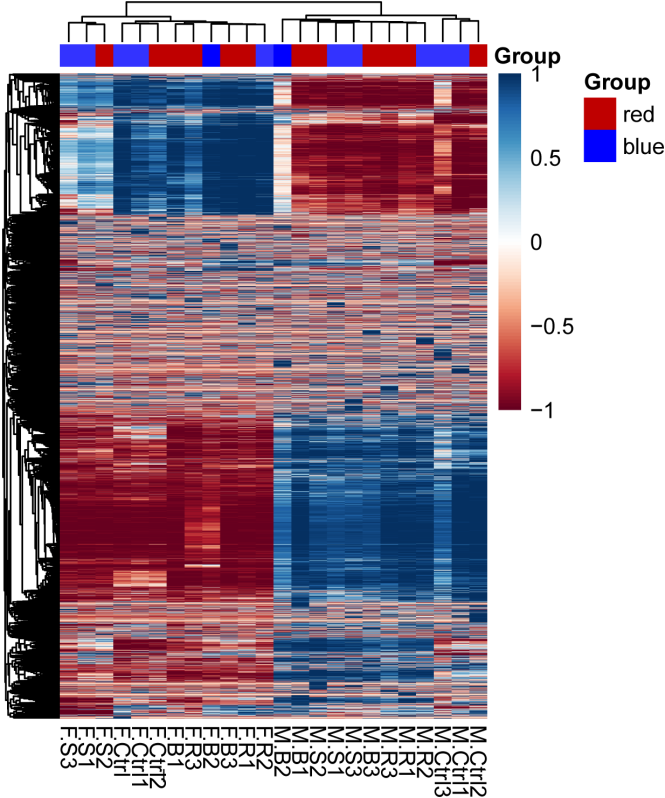


S4Fig. 6 Clustered heat map analysis The horizontal axis represents groups, and the vertical axis represents clustering. The redder the color, the greater the number of up-regulated genes; the bluer the color, the greater the number of down-regulated genes.

p<0.0001).

**volcano plot**

In female fruit flies, the PF group compared to the Control group had 417 up-regulated genes and 472 down-regulated genes (Fig. 7a). the ESP group compared to the PF group had 22 up-regulated genes and 23 down-regulated genes (Fig. 7c). the SSP group compared to the PF group had 393 up-regulated genes and 287 down-regulated genes (Fig. 7d).

In male fruit flies, the PF group compared to the Control group had 341 up-regulated genes and 237 down-regulated genes (Fig. 7b). The ESP group compared to the PF group had 79 up-regulated genes and 149 down-regulated genes (Fig. 7e). The SSP group compared to the PF group had 89 up-regulated genes and 207 down-regulated genes (Fig. 7f)


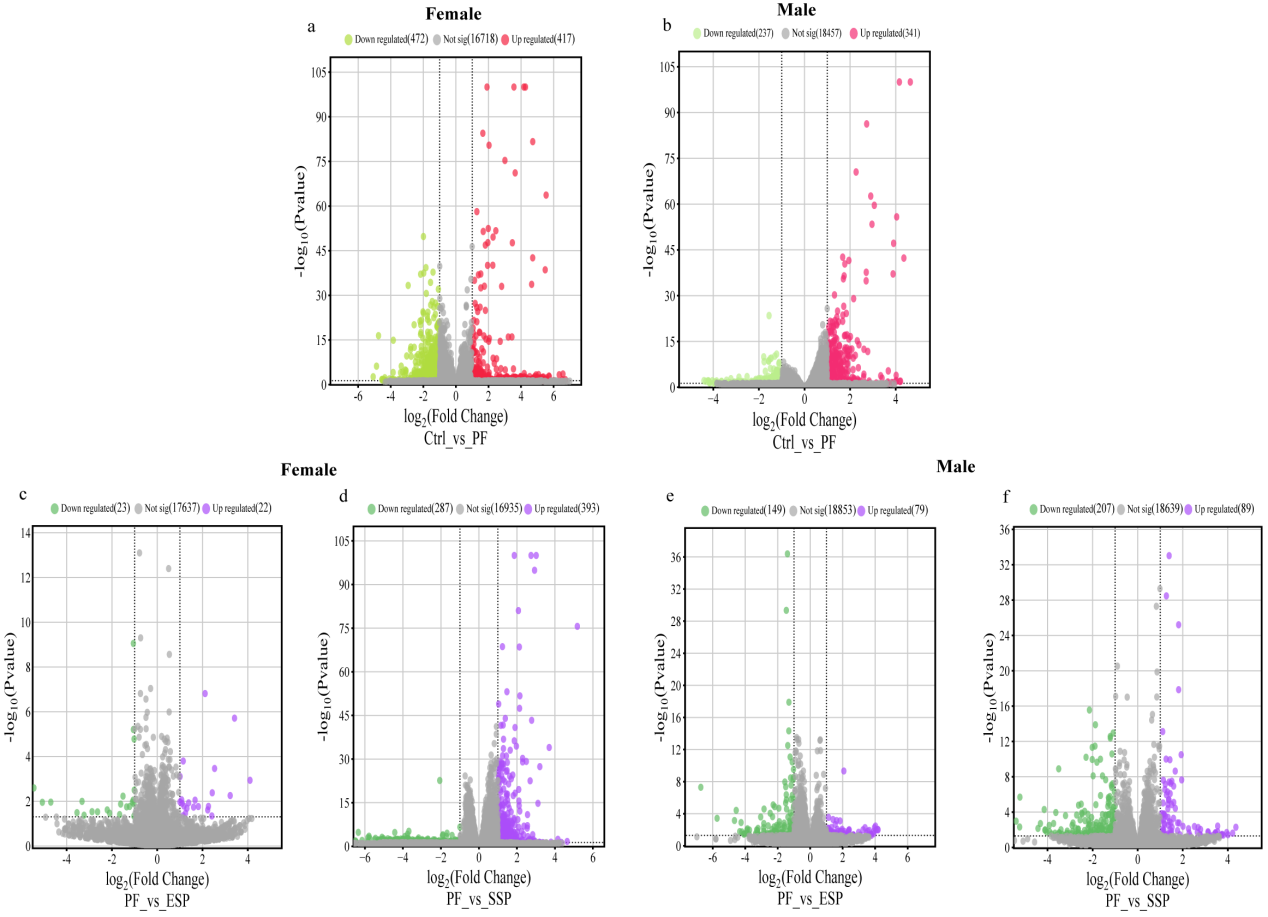


S4Fig. 7 The volcano plots of up-regulated and down-regulated gene expression are as follows a, c, d represent the up-regulated and down-regulated gene expression in female *Drosophila* when comparing the PF group to the Control group (Control_vs_PF), the ESP group to the PF group (PF_vs_ESP), and the SSP group to the PF group (PF_vs_SSP). b, e, f represent the up-regulated and down-regulated gene expression in male *Drosophila*.

**GO enrichment**

the PF group, compared to the Control group, primarily influenced biological processes such as sensory perception of chemical stimulus, innate immune response, defense response, response to pheromone, and response to bacterium. In the ESP group compared to the PF group, there was a decrease in gene expression related to the glycogen catabolic process and cellular detoxification of aldehyde. In the SSP group, compared to the PF group, there was a reduction in gene expression associated with the glycolytic process and innate immune response(S4 Fig.8)


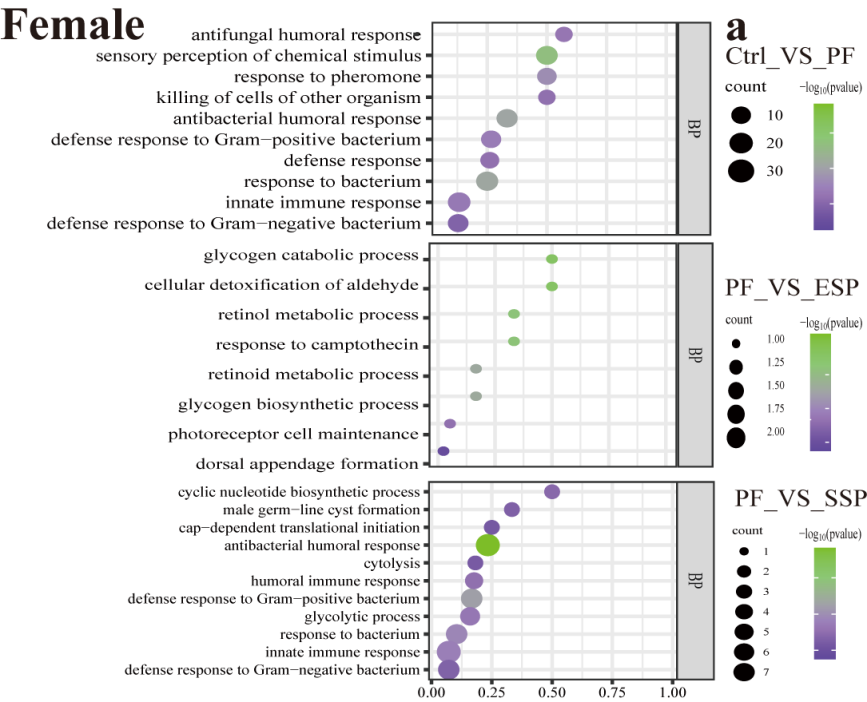


S4 Fig.8 a GO enrichment analysis, down-regulated gene changes in biological processes in Female *Drosophila*

a PF shows down-regulated expression compared to Control, ESP shows down-regulated expression compared to FP, SSP shows down-regulated expression compared to FP.


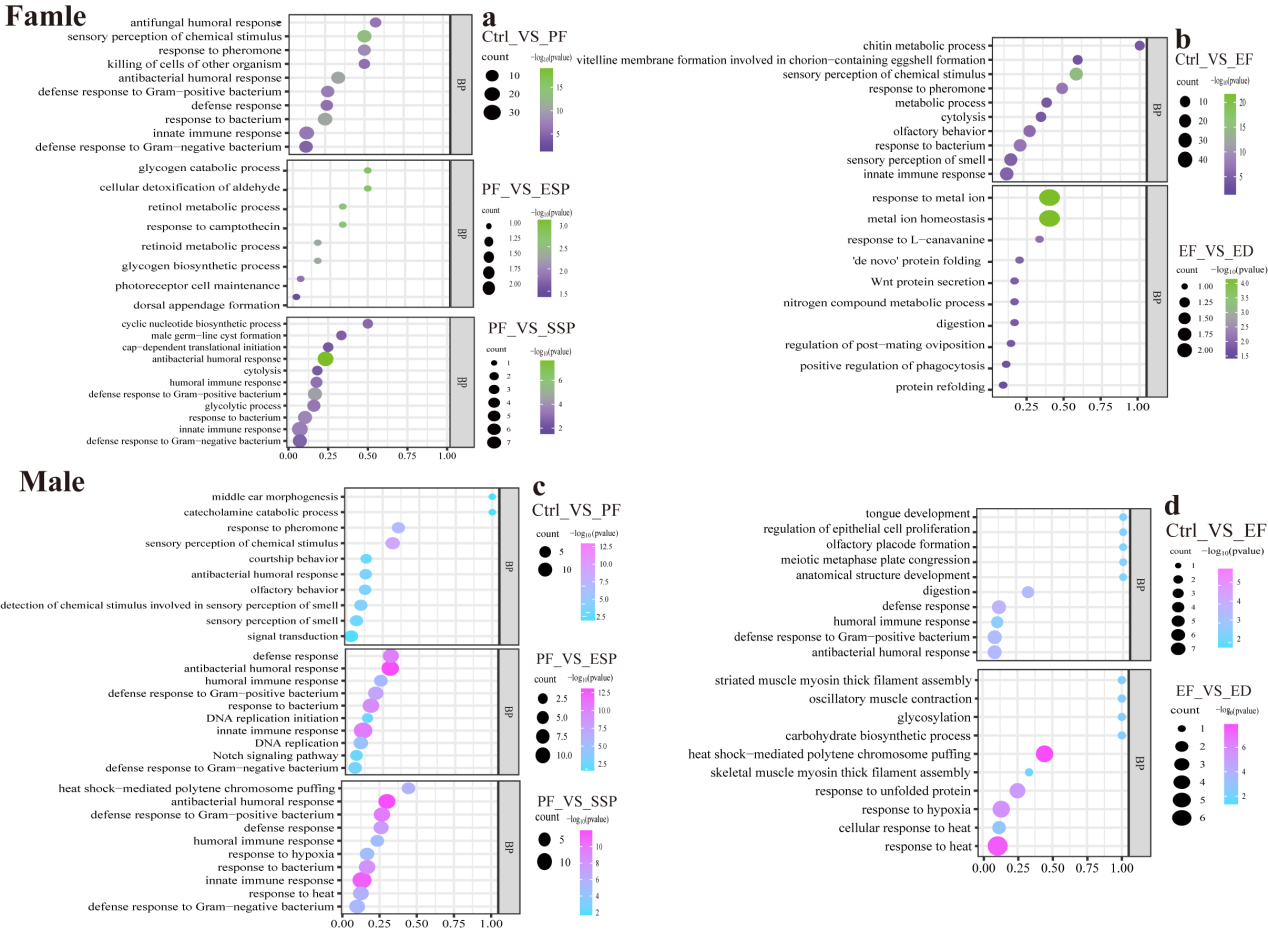


S4 Fig.8 c GO enrichment analysis, changes in down-regulated genes under biological processes
c PF shows down-regulated expression compared to Control, ESP shows down-regulated expression compared to FP, SSP shows down-regulated expression compared to FP.


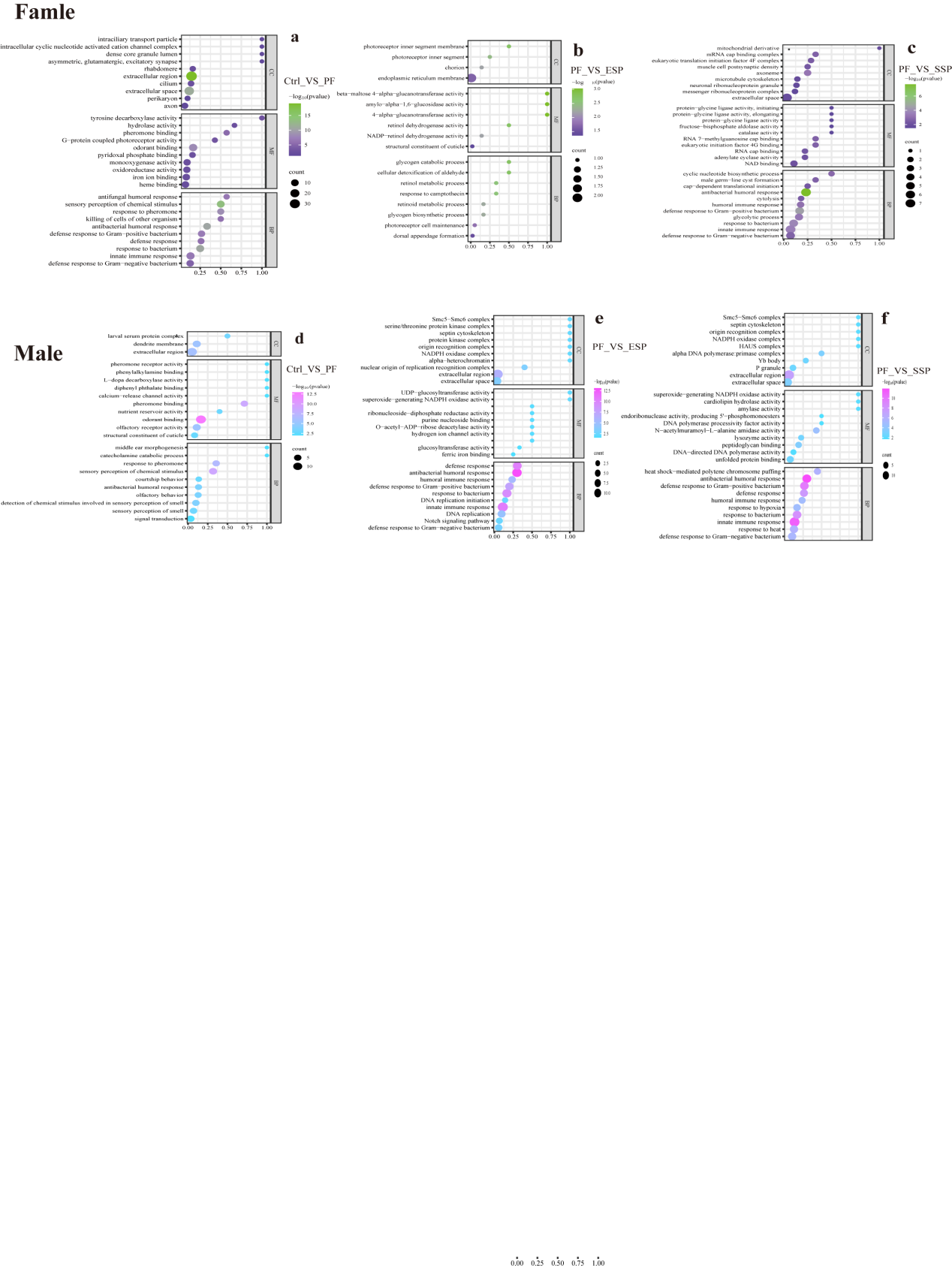


S4 Fig.8 d GO enrichment CC,MF,BP

**Supplementary 5**


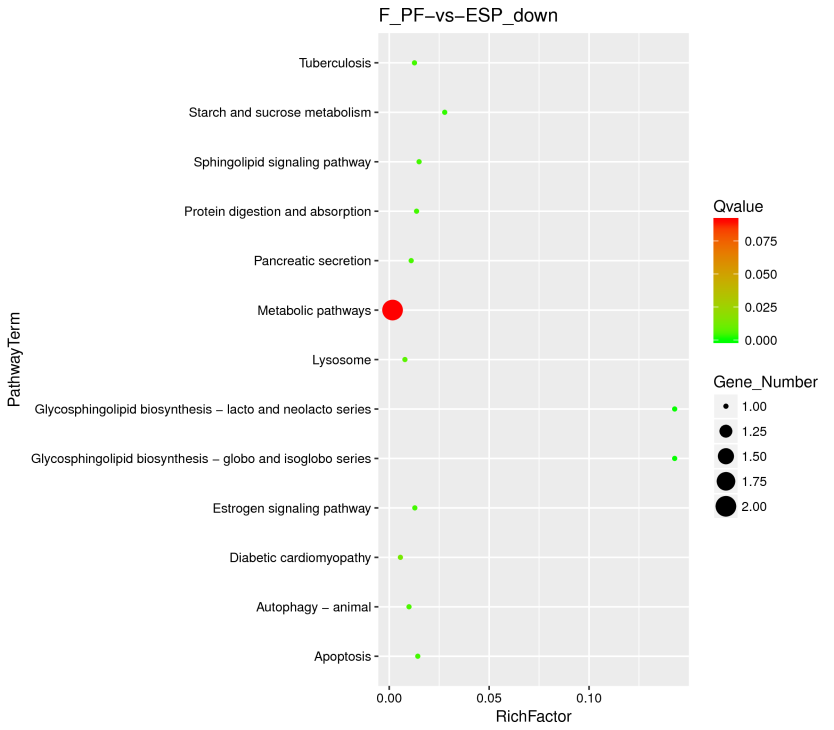


S5 Fig 1a

In male Drosophila, compared to the Control group, the PF group is mainly enriched in the Longevity regulating pathway−worm, Platinum drug resistance, Drug metabolism − cytochrome P450, and other pathways (S5 Fig. 2 c). Compared to the PF group, the ESP group is mainly enriched in the PPAR signaling pathway and Lysosome pathway (S5 Fig. 2 d). Compared to the PF group, the SSP group is mainly enriched in Toxoplasmosis, Spliceosome, Protein processing in the endoplasmic reticulum, and other pathways (S5 Fig. 2e).


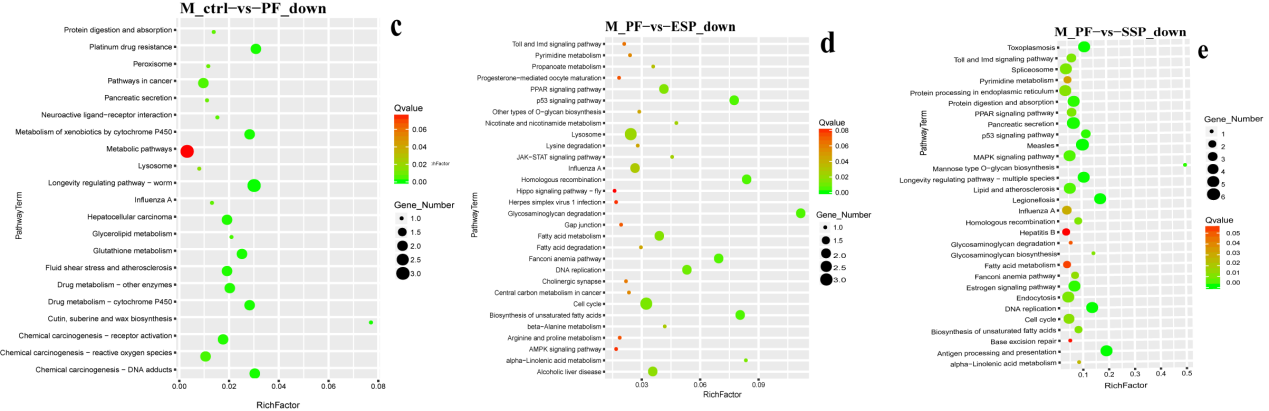


S4 Fig 2 c,d,e

In male Drosophila, the PF group, compared to the Control group, is mainly enriched in Metabolism of xenobiotics by cytochrome P450 and Chemical carcinogenesis-DNA adducts pathways(S5 Fig. 3d). The ESP group, compared to the PF group, is mainly enriched in Thiamine metabolism and Folate biosynthesis pathways(S5 Fig. 3e). The SSP group, compared to the PF group, is mainly enriched in Protein digestion and absorption and DNA replication pathways(S5 Fig. 3f).


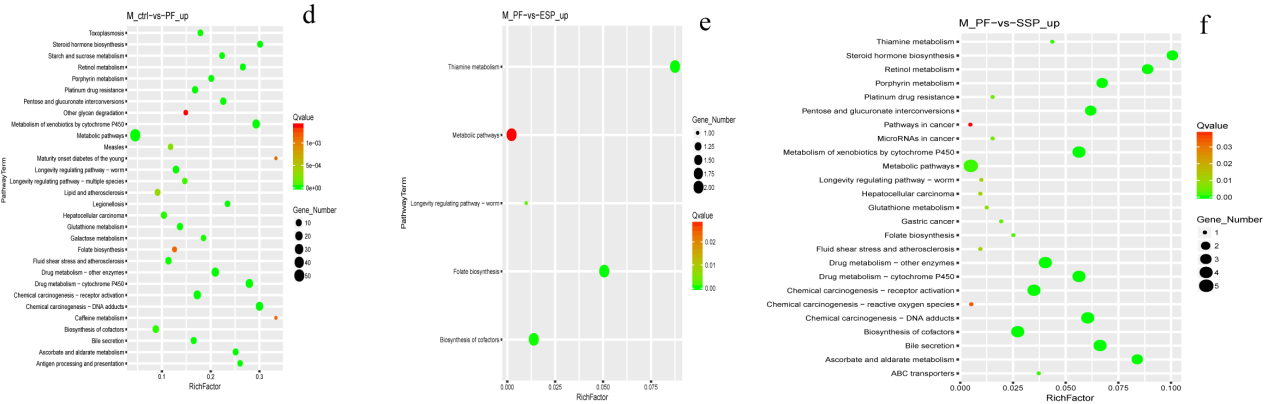


S5 Fig 3 d,e,f

**Supplementary 6**

**Table1**

| Ingredients | MW(g/mol) | SMILES | No. |
| --- | --- | --- | --- |
| p-Hydroxybenzaldehyde | 122.12 | C1=CC(=CC=C1C=O)O | 1 |
| 3,4-Dihydroxybenzaldehyde | 138.12 | O=CC1=CC=C(O)C(O)=C1 | 2 |
| 3-hydroxybenzaldehyde | 122.12 | C1=CC(=CC(=C1)O)C=O | 3 |
| Hydroquinone | 110.11 | C1=CC(=CC=C1O)O | 4 |
| Methylisoeugenol | 178.23 | C1(=CC(=CC=C1OC)/C=C/C)OC | 5 |
| 3-Hydroxy-4-methoxybenzaldehyde | 152.15 | COC1=C(C=C(C=C1)C=O)O | 6 |
| Isoeugenol | 164.2 | C1(=C(C=CC(=C1)/C=C/C)O)OC | 7 |
| (E)-3-(4-hydroxyphenyl)prop-2-enoic acid | 164.16 | C1=CC(=CC=C1/C=C/C(=O)O)O | 8 |
| pyrocatechol | 110.11 | C1=CC=C(C(=C1)O)O | 9 |
| 2,4-ditert-butylphenol | 206.32 | CC(C)(C)C1=CC(=C(C=C1)O)C(C)(C)C | 10 |
| 5-methyl-2-propan-2-ylphenol | 150.22 | CC1=CC(=C(C=C1)C(C)C)O | 11 |
| 4-hydroxy-3-methoxybenzaldehyde | 152.15 | COC1=C(C=CC(=C1)C=O)O | 12 |
| 2-methyl-5-propan-2-ylphenol | 150.22 | CC1=C(C=C(C=C1)C(C)C)O | 13 |
| 2-methoxy-4-prop-2-enylphenol | 164.2 | COC1=C(C=CC(=C1)CC=C)O | 14 |
| 2-methoxy-4-[(E)-prop-1-enyl]pheno | 164.2 | C/C=C/C1=CC(=C(C=C1)O)OC | 15 |
| 4-hydroxy-3-methoxybenzoic acid | 168.15 | COC1=C(C=CC(=C1)C(=O)O)O | 16 |
| 3,4-dimethoxybenzaldehyde | 166.17 | COC1=C(C=C(C=C1)C=O)OC | 17 |
| 4-hydroxy-3,5-dimethoxybenzaldehyde | 182.17 | COC1=CC(=CC(=C1O)OC)C=O | 18 |
| ethyl 4-hydroxybenzoate | 166.17 | CCOC(=O)C1=CC=C(C=C1)O | 19 |
| 4-ethenyl-2-methoxyphenol | 150.17 | COC1=C(C=CC(=C1)C=C)O | 20 |
| 1-(2-hydroxy-5-methylphenyl)ethanone | 150.17 | CC1=CC(=C(C=C1)O)C(=O)C | 21 |
| 2-methoxy-4-prop-2-enylphenol | 164.2 | COC1=C(C=CC(=C1)CC=C)O | 22 |

PF


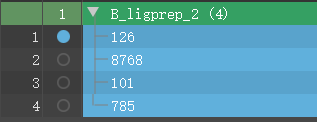


(1-4) corresponds to the components of No1-4 in table1, which corresponds to B-126,B-8768.... B-785.

ESP SSP


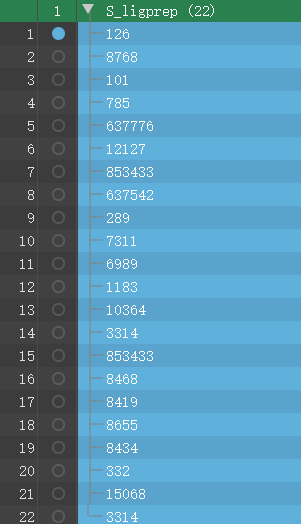

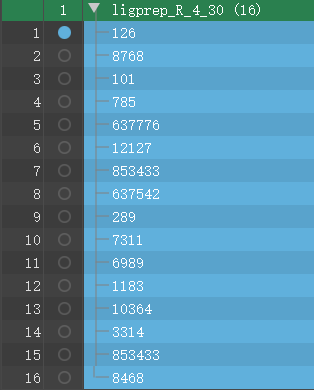


(1-16) corresponds to the components of No1-16 in table1, which corresponds to R-126,R-8768.... R-8468.

(1-22) corresponds to the components of No1-22 in table1, corresponding to S-126,S-8768.... S-3314.

| group | B | B | B | B | R | R | R | R | R | R | R | R | R | R | R | R | R | R | R |
| --- | --- | --- | --- | --- | --- | --- | --- | --- | --- | --- | --- | --- | --- | --- | --- | --- | --- | --- | --- |
| compounds | B-126 | B-101 | B-8768 | B-785 | R-7311 | R-6989 | R-10364 | R-12127 | R-126 | R-101 | R-8468 | R-853433 | R-637776 | R-289 | R-8768 | R-785 | R-1183 | R-637542 | R-3314 |
| LuxA_A | -5.895 | -5.864 | -5.566 | -5.019 | -6.677 | -6.49 | -6.476 | -6.306 | -5.984 | -5.867 | -5.855 | -5.82 | -5.639 | -5.032 | -5.566 | -5.019 | -5.733 | -5.222 | -5.125 |
| LuxA_C | -4.939 | -4.4 | -5.369 | -4.233 | -5.887 | -5.619 | -5.474 | -5.621 | -4.85 | -4.397 | -5.481 | -4.723 | -5.739 | -5.571 | -5.262 | -4.232 | -4.833 | -4.925 | -4.83 |
| LuxB_B | -5.581 | -5.139 | -6.014 | -5.62 | -6.76 | -5.25 | -6.054 | -5.441 | -5.492 | -5.135 | -5.027 | -5.256 | -5.349 | -4.835 | -5.907 | -5.619 | -5.026 | -4.229 | -4.727 |
| LuxB_D | -5.176 | -5.822 | -5.305 | -5.62 | -6.542 | -6.17 | -6.234 | -5.931 | -5.087 | -5.819 | -4.908 | -5.761 | -6.145 | -4.772 | -5.198 | -5.62 | -5.637 | -4.542 | -5.218 |
| LuxC | -4.996 | -5.819 | -5.240 | -5.562 | -5.878 | -4.847 | -6.015 | -4.896 | -4.996 | -5.819 | -4.827 | -6.181 | -5.626 | -4.737 | -5.240 | -5.562 | -4.844 | -7.052 | -4.28 |
| LuxD | -5.771 | -5.939 | -6.286 | -6.114 | -4.911 | -5.97 | -6.437 | -5.449 | -5.771 | -5.939 | -5.612 | -6.354 | -5.714 | -5.945 | -6.286 | -6.286 | -6.059 | -4.985 | -5.138 |
| LuxE | -5.774 | -6.842 | -5.366 | -5.819 | -4.5 | -4.968 | -6.291 | -5.200 | -5.774 | -6.842 | -6.012 | -5.875 | -6.243 | -5.545 | -5.366 | -5.819 | -5.832 | -4.25 | -5.642 |
| LuxG | -6.196 | -6.761 | -7.134 | -6.253 | -4.215 | 0 | -4.159 | -6.069 | -6.196 | -6.761 | -7.115 | -6.213 | -5.270 | -6.447 | -7.134 | -6.253 | -6.288 | -7.487 | -6.307 |

**Supplementary 7**

| group | S | S | S | S | S | S | S | S | S | S | S | S | S | S | S | S | S | S | S | S |
| --- | --- | --- | --- | --- | --- | --- | --- | --- | --- | --- | --- | --- | --- | --- | --- | --- | --- | --- | --- | --- |
| compounds | S-7311 | S-6989 | S-10364 | S-12127 | S-8419 | S-126 | S-101 | S-8468 | S-8655 | S-853433 | S-1183 | S-637776 | S-8768 | S-289 | S-785 | S-15068 | S-637542 | S-8434 | S-3314 | S-332 |
| LuxA_A | -6.677 | -6.49 | -6.476 | -6.302 | -5.96 | -5.895 | -5.864 | -5.855 | -5.843 | -5.82 | -5.733 | -5.639 | -5.566 | -5.032 | -5.019 | -5.328 | -5.222 | -5.334 | -5.125 | -5.364 |
| LuxA_C | -5.887 | -5.619 | -5.474 | -5.621 | -5.769 | -4.85 | -4.397 | -5.481 | -4.257 | -4.723 | -4.061 | -5.739 | -3.287 | -5.571 | -4.232 | -3.563 | -4.925 | -4.036 | -4.83 | -4.8 |
| LuxB_B | -6.76 | -5.25 | -6.054 | -5.441 | -5.596 | -5.492 | -5.135 | -5.027 | -5.539 | -5.256 | -5.026 | -5.349 | -5.907 | -4.835 | -5.619 | -3.164 | -4.229 | -5.023 | -4.727 | -4.879 |
| LuxB_D | -6.542 | -6.17 | -6.234 | -5.931 | -6.156 | -5.087 | -5.819 | -4.908 | -4.797 | -5.761 | -5.637 | -6.145 | -5.198 | -4.772 | -5.62 | -6.057 | -4.542 | -1.319 | -5.218 | -5.093 |
| LuxC | -5.878 | -4.847 | -6.015 | -4.896 | -5.138 | -4.996 | -5.819 | -4.827 | -5.540 | -6.181 | -4.844 | -5.626 | -5.240 | -4.737 | -5.562 | -5.189 | -7.052 | -4.017 | -4.28 | -4.462 |
| LuxD | -4.911 | -5.97 | -6.437 | -5.449 | -5.980 | -5.771 | -5.939 | -5.612 | -5.494 | -6.354 | -6.059 | -5.714 | -6.286 | -5.945 | -6.114 | -5.778 | -4.985 | -5.827 | -5.138 | -5.311 |
| LuxE | -4.5 | -4.968 | -6.291 | -5.200 | -4.461 | -5.774 | -6.842 | -6.012 | -5.044 | -5.875 | -5.832 | -6.243 | -5.366 | -5.545 | -5.819 | -5.055 | -4.25 | -4.846 | -5.642 | -5.561 |
| LuxG | -4.215 | 0 | -4.159 | -6.069 | -6.320 | -6.196 | -6.761 | -7.115 | -5.977 | -6.213 | -6.288 | -5.270 | -7.134 | -6.447 | -6.253 | -6.896 | -7.487 | -6.647 | -6.307 | -5.542 |


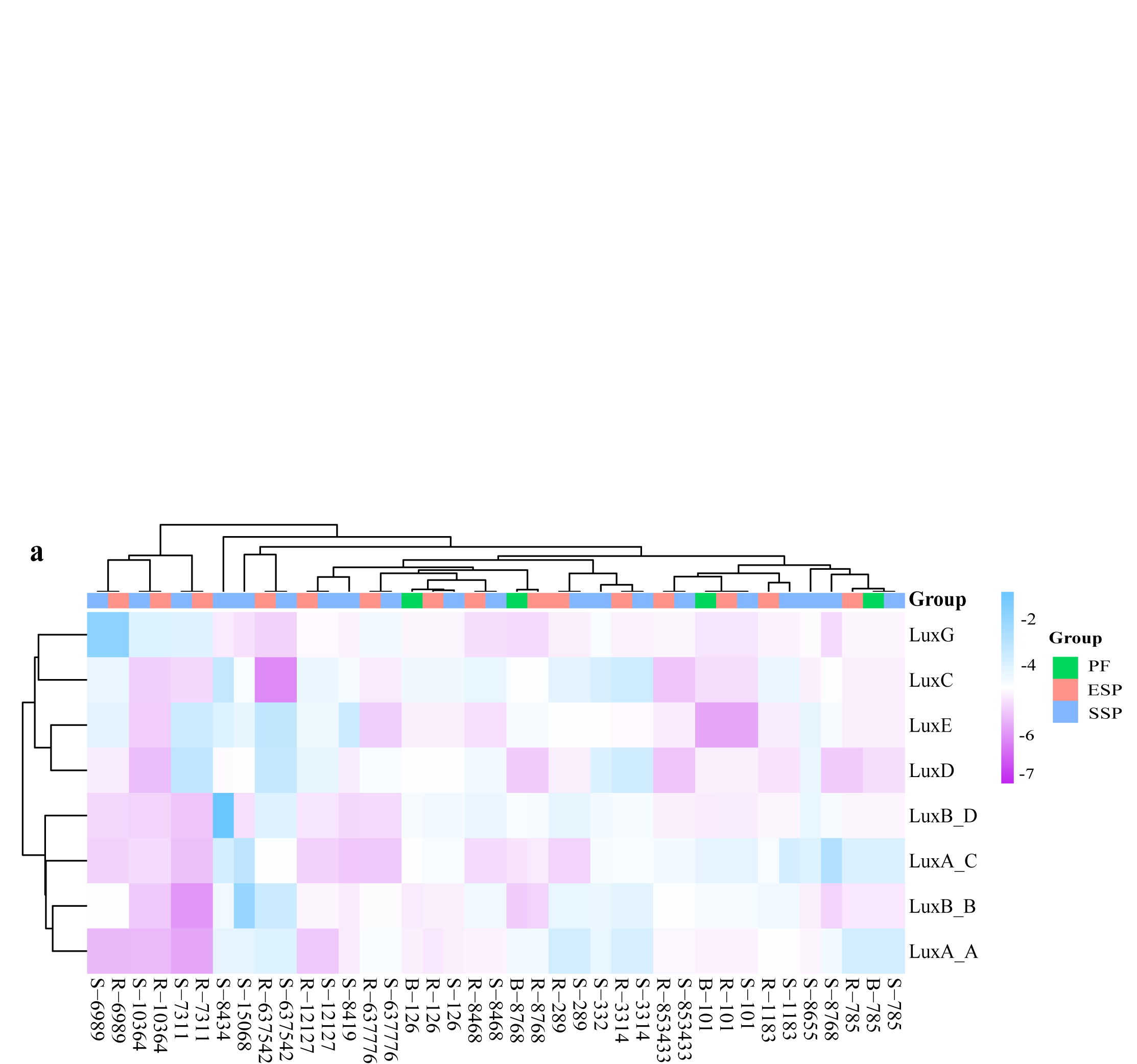


S7 Fig.1 Clustering analysis for proteins with ligands a Clustering of components in PF, ESP, SSP groups with proteins LuxA_A, LuxA_C, LuxB_B, LuxB_D, LuxC, LuxD, LuxE, LuxG. (B belongs to the components of PF, R belongs to the components of ESP, S belongs to the components of SSP. If the numerical identifiers of the components are the same, it indicates that they are the same component).

**Supplementary 8**

**PF**

**
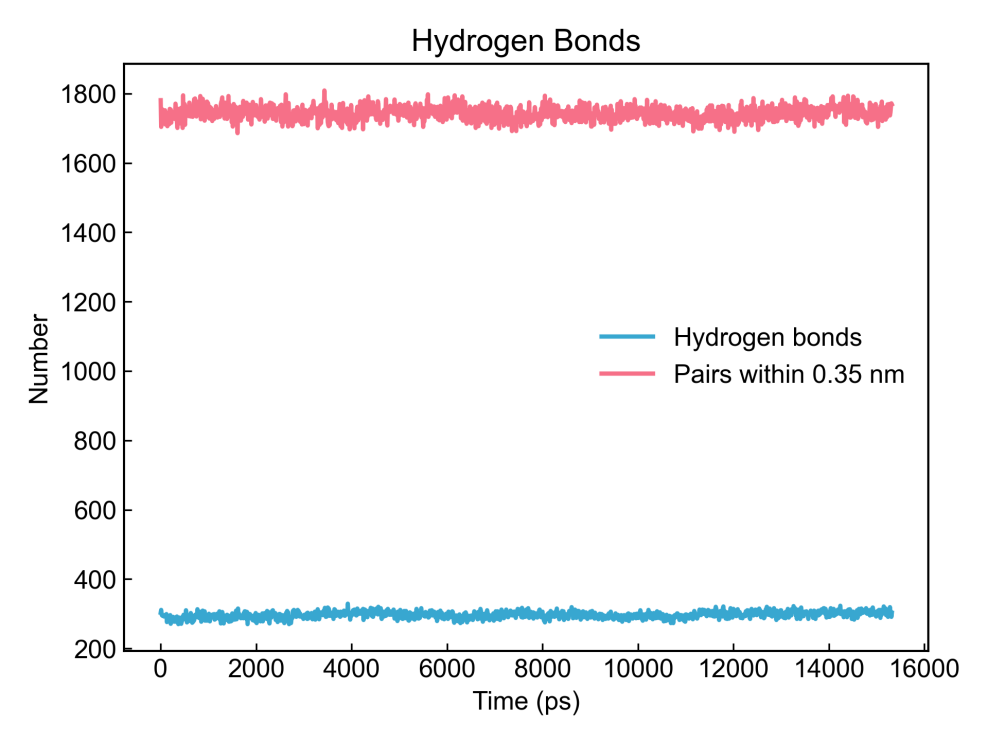
**

**ESP,SSP**

**
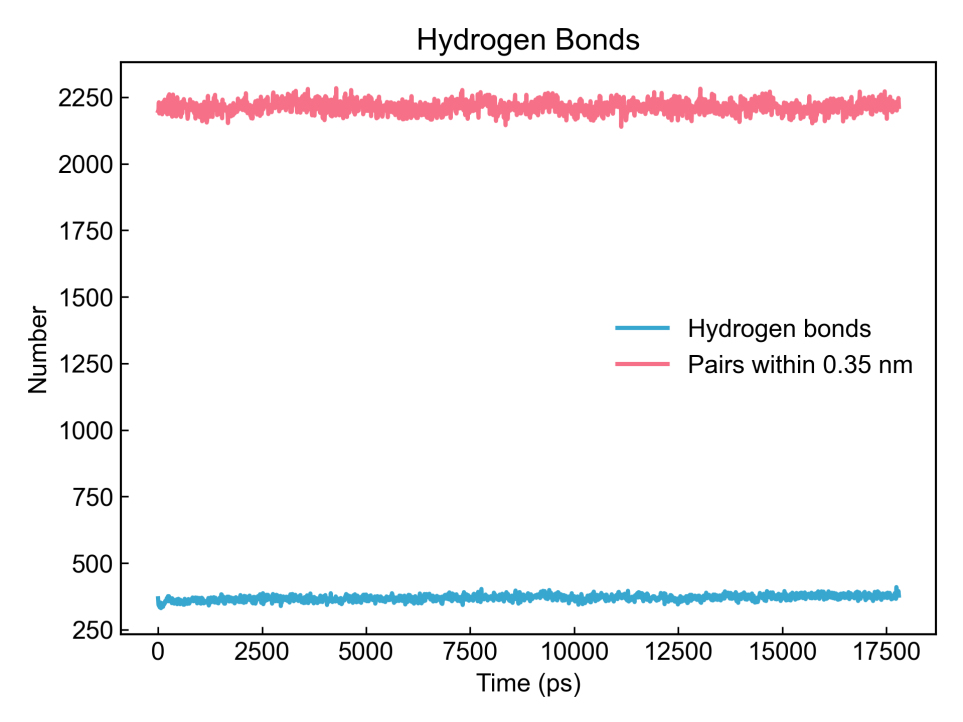
**

**(**https://github.com/ZhangChengCADEN/Supply/tree/main**)**
